# Lin28A promotes IRF6-regulated aerobic glycolysis in glioma cells by stabilizing SNHG14

**DOI:** 10.1038/s41419-020-2650-6

**Published:** 2020-06-11

**Authors:** Jinjing Lu, Xiaobai Liu, Jian Zheng, Jian Song, Yunhui Liu, Xuelei Ruan, Shuyuan Shen, Lianqi Shao, Chunqing Yang, Di Wang, Heng Cai, Shuo Cao, Yixue Xue

**Affiliations:** 1 Department of Neurobiology, School of Life Sciences, China Medical University, 110122 Shenyang, China; 2 Key Laboratory of Cell Biology, Ministry of Public Health of China, China Medical University, 110122 Shenyang, China; 3 Key Laboratory of Medical Cell Biology, Ministry of Education of China, China Medical University, 110122 Shenyang, China; 4 Department of Neurosurgery, Shengjing Hospital of China Medical University, 110004 Shenyang, China; 5 Liaoning Clinical Medical Research Center in Nervous System Disease, 110004 Shenyang, China; 6 Key Laboratory of Neuro-Oncology in Liaoning Province, 110004 Shenyang, China

**Keywords:** CNS cancer, Long non-coding RNAs

## Abstract

Warburg effect is a hallmark of cancer cells, wherein glycolysis is preferred over oxidative phosphorylation even in aerobic conditions. Reprogramming of glycometabolism is especially crucial for malignancy in glioma. RNA-binding proteins and long noncoding RNAs are important for aerobic glycolysis during malignant transformation. Thus, we determined the expression and function of RNA-binding protein Lin28A, long noncoding RNA SNHG14, and transcription factor IRF6 in human glioma cells to elucidate the mechanism(s) underlying their role in glycolysis. Quantitative real-time polymerase chain reaction and western blotting showed that Lin28A and SNHG14 were overexpressed and IRF6 was downregulated in glioma. Depleting Lin28A from cells decreased the stability and expression of SNHG14. Furthermore, depleting SNHG14 reduced IRF6 mRNA degradation by targeting its 3′ untranslated region and inhibiting STAU1-mediated degradation, thereby increasing the expression of IRF6. PKM2 is an important enzyme in aerobic glycolysis, and GLUT1 is the primary transporter that facilitates glucose uptake. IRF6 inhibited the transcription of PKM2 and GLUT1, thereby impairing glycolysis and cell proliferation and inducing apoptosis in glioma. Notably, depleting Lin28A and SNHG14 and overexpressing IRF6 reduced the growth of xenograft tumors in vivo and prolonged the survival of nude mice. Taken together, our data revealed that the Lin28A/SNHG14/IRF6 axis is crucial for reprogramming glucose metabolism and stimulating tumorigenesis in glioma cells. Thus, targeting this axis might help in the development of a novel therapeutic strategy for glioma metabolism.

## Introduction

Glioma is the most common and lethal malignant primary brain tumor that is highly aggressive with a very poor prognosis. Despite combination treatments, including surgery, radiotherapy, and chemotherapy, patients with glioma have a median survival time of 15 months^1^. Glucose is the main source of energy in the central nervous system (CNS); reprogramming glucose metabolism to combat glioma is a promising strategy, since reprogramming of cellular metabolism is a hallmark of cancer cells^2^. In contrast to normal cells, tumor cells rely on glycolysis to metabolize glucose even in normoxic conditions; this is called the Warburg effect^3^. Although glycolysis is inefficient in supplying ATP, the acidic microenvironment and intermediate metabolites generated by glycolysis are essential for malignant proliferation in glioma^4–6^.

RNA-binding proteins (RBPs) are important for the onset and progression of malignancies. Lin28A is important for the differentiation and proliferation of cancer as well as regulating cell metabolism^7^. Lin28A is overexpressed in liver cancer^8^ and colorectal cancer^9^. Previous studies have confirmed that Lin28A enhances aerobic glycolysis in hepatocellular carcinoma^10^. Lin28 is also overexpressed in glioblastoma and stimulates cell proliferation^11^. However, the effect of Lin28A on glycolysis in glioma is unknown.

Long noncoding RNAs (lncRNAs: >200 nucleotides long) are involved in the development, progression, and metastasis of cancers. SNHG14 is overexpressed in renal clear cell carcinoma^12^, breast cancer^13^, and gastric cancer^14^, accelerates malignant behavior. Moreover, ischemic cerebral tissues exhibit increased levels of SNHG14 that further exacerbates neuron damage^15^. However, very little is known about the effect of SNHG14 on glycolysis in glioma.

Interferon regulatory factor 6 (IRF6) is a member of the IRF family of transcription factors^16^. IRF6 is low-expressed and suppresses proliferation and differentiation in squamous cell carcinomas, such as nasopharyngeal and esophageal cancer, among others^16–18^. IRF6 induces apoptosis in the cortical neurons of the CNS^19^. However, the expression and function of IRF6 in glioma remain unclear.

Glucose transporters (GLUTs) facilitate glucose transporting into cells, which is the first rate limiting step in glycolysis. GLUT1, primarily facilitated glucose across the blood–brain barrier, is highly expressed in glioma cells and tissues wherein it stimulates proliferation and invasion^20^. Pyruvate kinase isozyme type M2 (PKM2) is a key enzyme in aerobic glycolysis^21–23^. Studies have confirmed that PKM2 contributes to the Warburg effect and tumorigenesis in glioma^24^.

We analyzed the expression and function of Lin28A, SNHG14, IRF6, GLUT1, and PKM2 and their underlying mechanisms in the tumorigenesis of glioma. We hypothesized that interactions within the Lin28A/SNHG14/IRF6 axis may be responsible for reprogramming glucose metabolism, thereby stimulating the development and progression of glioma. We hope our findings might indicate a novel therapeutic strategy for glioma metabolism.

## Materials and methods

### Clinical tissues

Studies were approved by the institutional ethics committee of Shengjing Hospital of China Medical University; consent was obtained from all subjects. Specimen were obtained from Neurosurgery Department of Shengjing Hospital of China Medical University, immediately frozen and preserved in liquid nitrogen after surgical resection. Glioma tissues were classified into low-grade glioma tissues (LGGTs; grade I–II), and high-grade glioma tissues (HGGTs; grade III–IV), according to the WHO 2007, by two experienced neuropathologists. Normal brain tissues (NBTs) were collected from patients’ fresh autopsy material (donation from individuals who died in traffic accident and confirmed to be free of any prior pathologically detectable conditions) and were used as negative control.

### Cell culture

Human glioma cell lines (U87, U251) and human embryonic kidney (HEK) 293T cells were maintained in Dulbecco modified Eagle medium (DMEM) (SH30022.01B, Hyclone) supplemented with 10% fetal bovine serum (10099141, Gibco, USA), cell lines were purchased from the Chinese Academy of Medical Sciences (Beijing, China). Normal human astrocyte (NHA) cells were purchased from Sciencell Research Laboratories (Carlsbad, CA, USA) and were cultured in RPMI-1640 medium (SH30809.01, Hyclone). All cells were cultured in a humidified incubator at 37 °C with 5% CO_2_.

### RNA isolation and quantitative real-time PCR (qRT-PCR)

Total RNA was extracted from human tissue samples and HA, U87, U251 cells using trizol reagent (15596026, Life Technologies Corporation, Carlsbad, CA). RNA quality and concentration were measured with Nanodrop Spectrophotometer (ND-100, Thermo, Waltham, MA). SNHG14, Lin28A mRNA, IRF6 mRNA and GAPDH mRNA abundance were detected using One-Step SYBR® PrimeScript^TM^ RT-PCR Kit (RR066A, TakaraBio, Shiga, Japan) in 7500 Fast RT-PCR System (Applied Biosystems, USA). The relative quantification (2^−^^△△Ct^) and were calculated using GAPDH as endogenous control. Primers are shown in Supplementary Table 1.

### Western blotting

Total proteins were gained after cells were lysed in RIPA buffer (P0013B, Beyotime, China,). Protein samples were subjected to SDS-PAGE and transferred to polyvinylidene difluoride membranes. After incubation in 5% nonfat milk, membranes were embraced by primary antibodies (Supplementary Table 2). Then incubated with horse radish peroxidase-conjugated secondary antibodies. Immunoblots were visualized with ECL Kit (P0018AS, Beyotime). Integrated density values were calculated using GAPDH as endogenous control.

### Cell transfections

Short hairpin RNAs (shRNAs) targeted to Lin28A (sh-Lin28A), SNHG14 (sh-SNHG14), IRF6 (sh-IRF6), STAU1 (sh-STAU1), UPF1 (sh-UPF1) and their control scrambled shRNA were synthesized. (GeneChem, Shanghai, China). Lin28A, SNHG14, IRF6 overexpressed plasmid and their respective nontargeting sequence (negative control, NC) were synthesized (GenePharma, Shanghai, China) (Supplementary Table 3). Cells were seeded and cultured to 50–70% confluence, then transfected with Lipofectamine 3000 reagent (L3000001, Life Technologies) according to the manufacturer’s instruction. The stable transfected cells were selected using G418 or puromycin. To avoid shRNA-mediated off target effects, we transfected silencing plasmids at multiple sites and tested transfection efficiency. Then, the shRNA with the best silencing efficiency was selected to further functional study. The depletion and overexpressions efficiency were measured by qRT-PCR and western blotting (Supplementary Figs. S1a–o, S2a–c).

### Cell proliferation assay

Cell Counting Kit-8 (CK04, Dojindo, Japan) assay was performed to measured cell proliferation according to the manufacturer’s protocol. The absorbance was measured at 450 nm with the SpectraMax M5 microplate reader (Molecular Devices).

### ECAR measurement

Extracellular acidification rate (ECAR) was measured according to the manufacturer’s protocol of XF glycolysis stress test kit (103020-100, Seahorse Bioscience, USA), using the XF24 Extracellular Flux Analyzer (Seahorse Bioscience, Billerica, MA, USA, kindly provided by the Experimental Teaching Center, School of Public Health, China Medical University). Cells were plated 50,000 cells/well in 24-well cell culture microplates (100777-004, Seahorse Bioscience) in 500 µl medium and incubated overnight at 37 °C under 5% CO_2_. A XF24 sensor cartridge (100850-001, Seahorse Bioscience) was hydrated overnight as well.

Initially, the cell was cultured in XF Base Medium (without glucose, supplemented with 2 mM glutamine, pH = 7.4), and incubated at 37 °C in a non-CO_2_ incubator to maintain glucose starvation conditions. The ECAR was measured under glucose starvation conditions (0–17 min, represented as nonglycolysis). Next, to determine the basal glycolysis rate, we added glucose with a final concentration of 10 mM. Glycolysis was induced and ECAR was measured three times again (26–45 min). For evaluating glycolysis capacity, oligomycin was subsequently injected. Oxidative phosphorylation was inhibited by oligomycin causing cells to completely rely on glycolysis for energy and increasing their acidic contents, thereby leading to a further increase in ECAR (52–72 min). Finally, we injected 2-deoxyglucose (2-DG) to competitively inhibit total glycolysis (78–96 min). Cell numbers were rechecked after measurement. Measurements were all normalized by cell numbers. Glycolysis was calculated by subtracting the nonglycolysis ECAR from the ECAR obtained after adding glucose. Glycolytic capacity was calculated by subtracting the nonglycolysis ECAR from ECAR obtained after adding oligomycin, and taken control group as 1.

### Lactate assay

Targeted cells were plated 20,000 cells per well into 96-well culture plate with 200 μl medium. The supernatants were collected after 48-h culturing. Lactate levels were measured using a lactate colorimetric assay read at 540 nm according to the manufacturer’s instructions (Jiancheng, China) and normalized to the cell numbers, taken control group as 1.

### Glucose uptake assay

Targeted cells were cultured in glucose-free DMEM for 16 h, and then incubated with high-glucose DMEM with 2-DG for an additional 20 min at 37 °C; the intracellular glucose levels were measured using a Glucose Uptake Assay (ab136955, Abcam, USA) according to the manufacturer’s instructions. Briefly, cells were lysed with extraction buffer, then freezed/thawed and heated at 85 °C for 40 min, and cooled on ice for 5 min. Supernatants were collected to wells, reaction mix A was added and incubated for 1 h at 37 °C, extraction buffer was added and heated to 90 °C for 40 min, cooled on ice for 5 min and reaction mix B was added and analyzed on microplate reader. Finally, the results normalized to the cell numbers, taken control group as 1.

### Apoptosis detection

Cell apoptosis was detected by staining with Annexin V-PE/7AAD (559763, BD Biosciences, USA) according to the manufacturer’s instructions. After washing with phosphate-buffered saline and centrifuging twice, cells were resuspended in Annexin-V binding buffer. They were stained with Annexin V-PE/7AAD and incubated for another 15 min at room temperature in a dark room. Cell samples were analyzed by flow cytometry (FACScan, BD Biosciences) to acquire the apoptotic fractions.

### Dual-luciferase reporter assay

Potential SNHG14 binding sites of IRF6 3′-UTR were predicted by bioinformatics tool Starbase (http://starbase.sysu.edu.cn/). The 3′-UTR fragment of the IRF6 gene with the theoretical SNHG14 binding site was constructed in pGL3 plasmid vector to form the reporter vector IRF6-3′-UTR-wild-type (IRF6-3′-UTR-Wt) (GenePharma). To mutate the putative binding site of SNHG14 in the 3′-UTR-containing vector, the sequence of the putative binding site was replaced as indicated and formed the IRF6-3′-UTR-mutated-type (IRF6-3′-UTR-Mut). The plasmid vector (wild-type fragments or mutated type fragments) was transfected into HEK 293T cells using Lipofectamine 3000. Relative luciferase activities were measured 48 h after transfection and firefly luciferase activity was normalized by renilla luciferase activity.

### Fluorescence in situ hybridization (FISH)

FISH assay was preformed according to the manufacturer’s instructions using the QuantiGene ViewRNA miRNA ISH cell assay kit (QVCM0001). Briefly, cells were fixed in 4% formaldehyde for 15 min and then washed with PBS. The fixed cells were treated with pepsin and dehydrated through ethanol. The air-dried cells were incubated further with FISH probe, followed by incubation with preamplifier mix, amplifier mix, and label probe mix at 40 °C for 30 min respectively.

For immunofluorescence analysis, cells were fixed with 4% formaldehyde followed by incubation with rabbit anti-Lin28A (16177-1-AP, Proteintech) followed by Alexa Fluor 488 anti-rabbit secondary antibody (A32731, Invitrogen) incubation. The nucleus was stained with DAPI (Invitrogen). The slides were visualized for immunofluorescence with an Olympus microscope.

### Chromatin immunoprecipitation (ChIP) assay

ChIP assay was conducted with Simple ChIP Enzymatic Chromatin IP Kit (#22188, Cell Signaling Technology, Danvers, Massachusetts, USA) according to the manufacturer’s protocol. Briefly, glioma cells were fixed with 1% formaldehyde and collected in cold lysis buffer. Two percent aliquots of lysates were used as an input control and the other immunoprecipitation samples were incubated with normal rabbit IgG or anti-IRF6 antibody. Immunoprecipitated DNA was amplified by PCR using their specific primers (Supplementary Table 4).

### Nascent RNA capture assay

Nascent RNA capture assay was performed using the Click-iT Nascent RNA Capture Kit (Invitrogen) following the manufacturer’s protocol. Briefly, cells were incubated in 5-ethymyl uridine (EU) and total RNA labeled with EU was isolated using Trizol reagent. Subsequently, EU-labeled RNA was biotinylated in a Click-iT reaction buffer and then captured using streptavidin magnetic beads.

### RNA half-life measurement

Actinomycin D (1 μg/ml) was added to block de novo RNA synthesis; total RNA was collected at indicated times and RNA expression was measured by qRT-PCR. The half-life of RNA was determined as the time required to reach 50% of the RNA levels before adding actinomycin D.

### RNA immunoprecipitation (RIP) assay

RIP assays were performed according to the instructions of RNA Immunoprecipitation (RIP) Kit (catalog bes5101, Bersin Bio^TM^). Briefly, cell lysate was incubated with RIP buffer containing magnetic beads conjugated with human anti-STAU1 antibody or negative control normal mouse IgG. Samples were incubated with proteinase K and immunoprecipitated RNA was isolated. Furthermore, RNA was purified from RNA-protein complexes, bound to the beads, and then was analyzed by qRT-PCR.

### RNA pulldown assay

In human embryonic kidney (HEK) 293T cells, according to the instructions of Pierce TM Magnetic RNA-Protein Pull-Down Kit(20164, Thermo), biotin-labeled SNHG14 or antisense RNA were synthesized and then incubated with the cell lysates for 4 h. The protein with biotin-labeled SNHG14 or antisense RNA was pulled down with streptavidin magnetic beads after incubation overnight. The generated bead-RNA-Protein compound was collected by low-speed centrifuge, and eluted through Handee spin columns. Subsequently, the bead compound was boiled in SDS buffer, and the retrieved proteins were detected by western blot.

### In vivo study

The 4-week-old BALB/C athymic nude mice were obtained from Cancer Institute of the Chinese Academy of Medical Science, and randomly divided into five groups: control, sh-Lin28A, sh-SNHG14, IRF6 and sh-Lin28A+sh-SNHG14 + IRF6 groups. For subcutaneous implantation, 3 × 10^5^ cells were injected subcutaneously under right axilla. Tumors were measured blindedly every 4 days, calculated by the formula: volume (mm^3^ = length × width^2^/2). The mice were sacrificed and tumors were isolated on the 44th day post inoculation. As for intracranial orthotopic inoculation, cells were implanted into the right striatum of mice stereotactic ally. The number of survived nude mice was recorded every day and survival analysis was conducted applying the Kaplan−Meier survival curve. Experiments were approved by the Ethics Committee of China Medical University.

### Statistical analysis

Data are presented as mean ± SD from at least three independent experiments. All statistical analyses were performed by SPSS 18.0 statistical software (IBM, New York, NY) with the Student’s *t* test (two tailed) or one-way analysis of variance. Survival analysis was evaluated using the Kaplan−Meier method and assessed using the log-rank test. Differences were considered statistically significant when *P* < 0.05.

## Results

### Lin28A was upregulated in glioma cell lines and tissues and knockdown of Lin28A inhibited glycolysis and proliferation

**Fig. 1:**
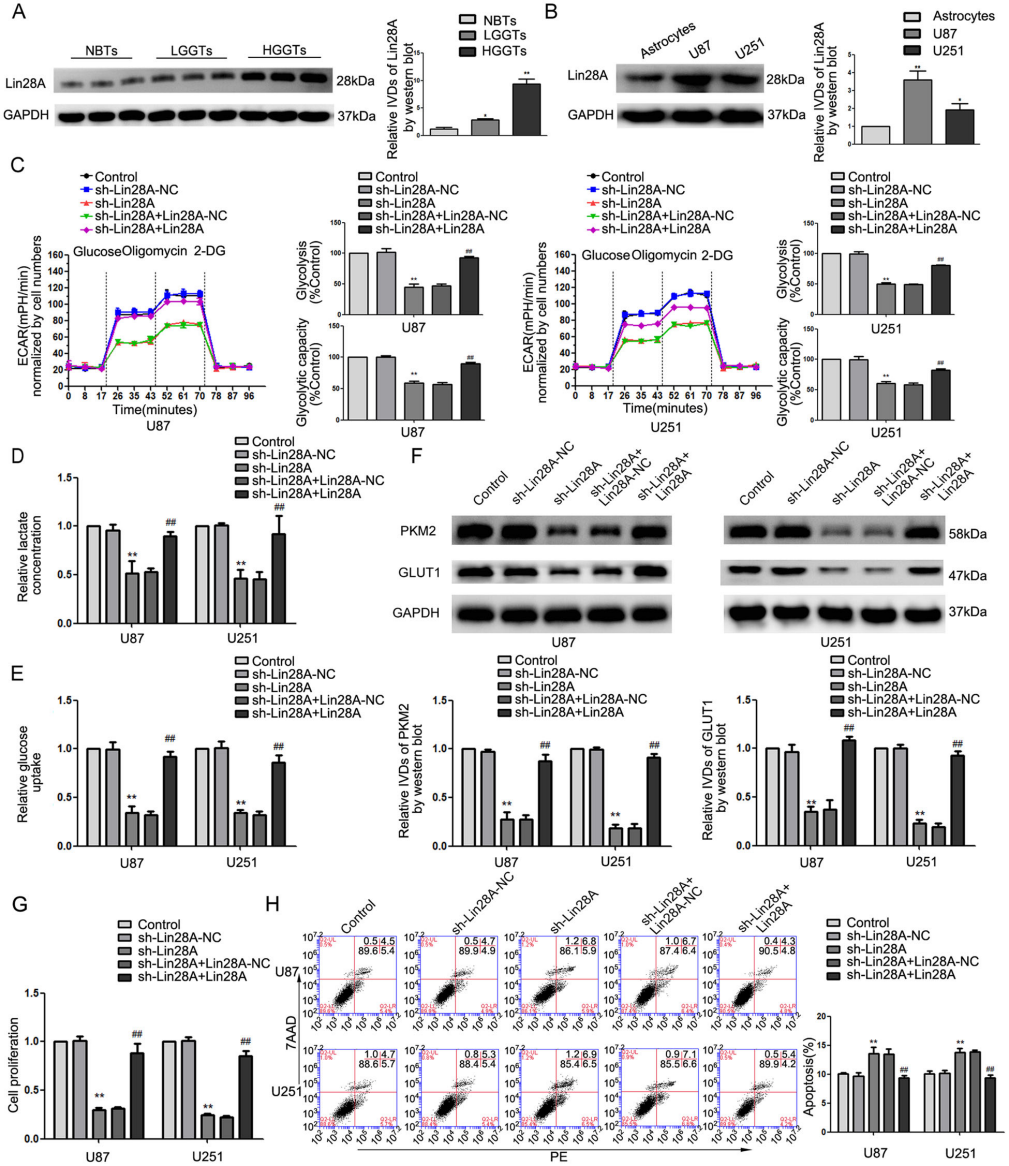
Lin28A was upregulated in glioma cell lines and tissues and knockdown of Lin28A inhibited glycolysis and proliferation. **a** Protein levels of Lin28A in normal brain tissues (NBTs), low-grade glioma tissues (WHO I-II), and high-grade glioma tissues (WHO III-IV). Data are presented as the mean ± SD (*n* = 3, each group). *P < 0.05, **P < 0.01 versus NBTs group. **b** Protein levels of Lin28A in normal human astrocytes (NHA), glioma cell lines (U87 and U251) cells. Data are presented as the mean ± SD (*n* = 3, each group). *P < 0.05, **P < 0.01 versus NHA group. **c** Extracellular acidification rate (ECAR) was measured to detect the effect of Lin28A depletion and re-expression of Lin28A in sh-Lin28A cells on glycolysis in glioma; glycolysis and glycolytic capacity were analyzed. **d**, **e** The lactate production and glucose uptake in response to Lin28A depletion and re-expression in U87 and U251 cells. **f** Expression of PKM2 and GLUT1 by western blot upon Lin28A depletion and re-expression. **g** CCK-8 assay was conducted to investigate the effect of Lin28A on proliferation. **h** Flow cytometry analysis to evaluate the effect of Lin28A on apoptosis. Data are presented as the mean ± SD (*n* = 3, each group). **P < 0.01 versus sh-Lin28A-NC group, ^##^*P* < 0.01 versus sh-Lin28A + Lin28A-NC group. One-way analysis of variance was used for statistical analysis.

We used the Oncomine database (http://www.oncomine.org) to evaluate the differential expression of Lin28A in glioblastoma and NBTs and generated a plot correlating overall survival with Lin28A expression. As shown in Supplementary Fig. S2d, compared to the expression of Lin28A in NBTs (*n* = 37), Lin28A was upregulated in glioblastoma brain tissues (*n* = 582). Patients with high expression of Lin28A had shorter survival periods than those with low Lin28A expression (Supplementary Fig. S2e). We used quantitative real-time PCR (qRT-PCR) and western blotting to detect Lin28A expression. Lin28A was overexpressed in glioma tissues (as compared to NBTs; Fig. 1a), U87, and U251 cells (as compared to NHA; Fig. 1b). We generated cell lines with stable Lin28A knockdowns and re-expression of Lin28A in knockdown cells to understand its function. Extracellular acidification rate (ECAR), specific for glycolytic acidification, was simultaneously measured by the Seahorse XF analyzer. Then glycolysis and glycolytic capacity were calculated (see the “Materials and methods” section). Figure 1c shows glycolysis and glycolytic capacity of Lin28A-depleted cells were decreased as compared to the control group and re-expressing Lin28A reversed this phenotype. The contrasting ECARs could be attributed to the difference in glycolytic tendencies in each group.

Glycolysis could be induced by increasing the glucose intake, and leads to accumulating lactate; therefore, we measured the production of lactate and glucose uptake in glioma. Lactate production and glucose uptake were significantly reduced in sh-Lin28A cells (Fig. 1d, e). Since PKM2 and GLUT1 are well-known regulators of glycolysis in tumors, we further investigate whether PKM2 and GLUT1 were involved in Lin28A-mediated reprogramming of glucose metabolism in glioma. PKM2 and GLUT1 significantly decreased in cells with sh-Lin28A (Fig. 1f). A cell counting kit-8 assay showed that inhibiting Lin28A suppressed the proliferation of glioma cells (Fig. 1g). Moreover, flow cytometry revealed that silencing Lin28A markedly increased apoptosis in the glioma cells (Fig. 1h). Re-expressing Lin28A in the sh-Lin28A (knockdown) cells reversed these phenotypes. Our results suggest that Lin28A is an oncogene that, when silenced, inhibits glycolysis, impairs proliferation, and enhances apoptosis in glioma cells.

### SNHG14 was upregulated in glioma and knockdown of SNHG14 suppressed glycolysis and proliferation

**Fig. 2:**
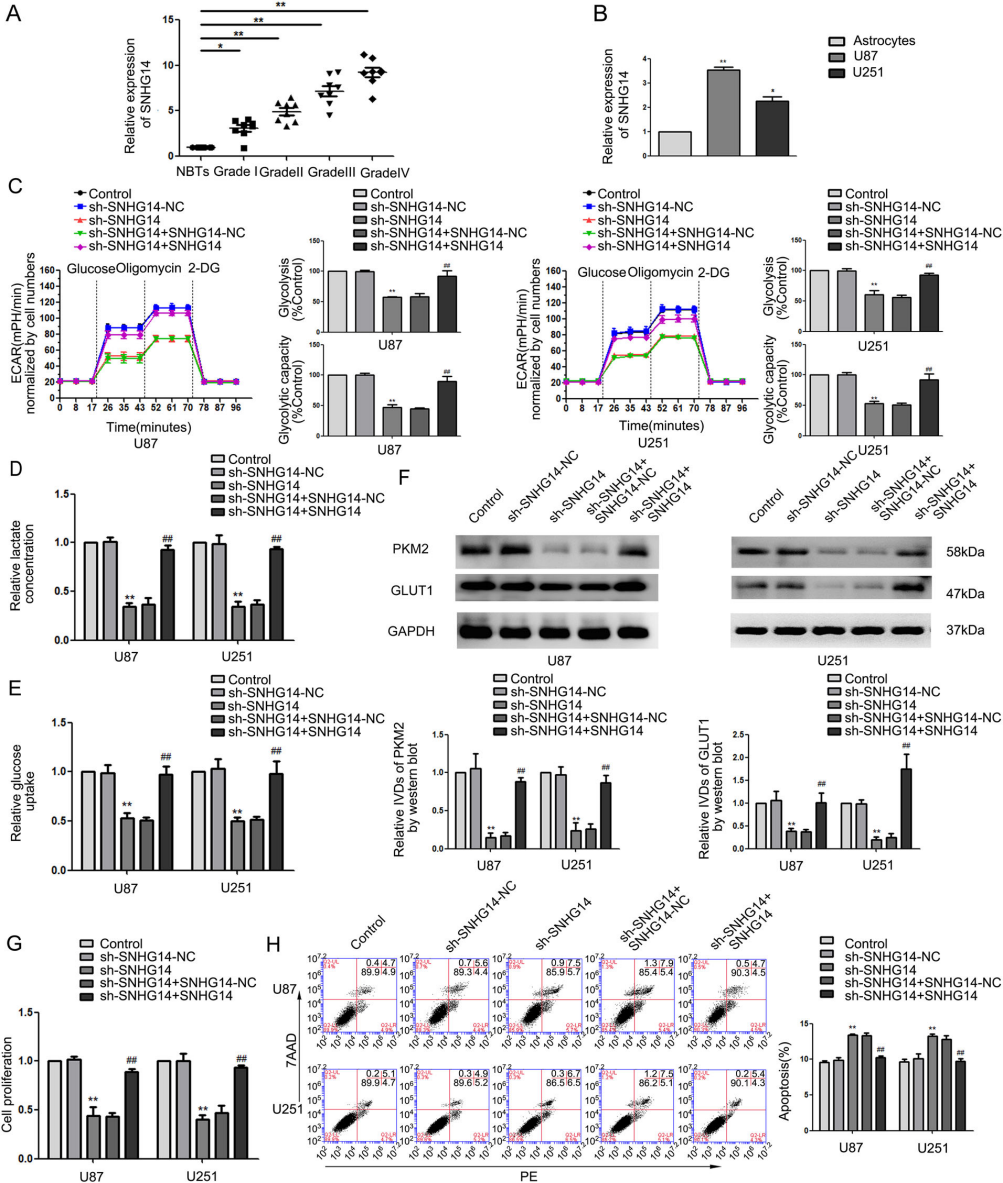
SNHG14 was upregulated in glioma and knockdown of SNHG14 suppressed glycolysis and proliferation. **a** The expression of SNHG14 in glioma tissues and NBTs. Data are presented as the mean ± SD (*n* = 8 in each group). *P < 0.05, **P < 0.01 versus NBTs group. **b** The expression of SNHG14 in NHA, U87 and U251. Data are presented as the mean ± SD (*n* = 3 in each group). *P < 0.05, **P < 0.01 versus NHA group. **c** ECAR was measured to detect the effect of SNHG14 depletion and re-expression on glycolysis. **d**, **e** The lactate production and glucose uptake in response to SNHG14 depletion and re-expression. **f** Expression of PKM2 and GLUT1 by western blot upon SNHG14 depletion and re-expression. **g** CCK-8 assay was conducted to investigate the effect of SNHG14 in cell proliferation. **h** Flow cytometry analysis was used to evaluate the function of SNHG14 in apoptosis. Data are presented as the mean ± SD (*n* = 3, each group). **P < 0.01 versus sh-SNHG14-NC group, ^##^*P* < 0.01 versus sh-SNHG14 + SNHG14-NC group. One-way analysis of variance was used for statistical analysis.

We performed lncRNA microarrays that showed SNHG14 was significantly downregulated in sh-Lin28A glioma cells (Supplementary Fig. S2f). As shown in Fig. 2a, SNHG14 was overexpressed in glioma tissues as compared to NBTs and further increased with the advancement in the grade of glioma. Expression of SNHG14 was higher in the U87 and U251 cell lines than that in NHA (Fig. 2b). We generated cell lines with stable knockdowns and re-expression of SNHG14 to understand its function. As shown in Fig. 2c, the ECAR of sh-SNHG14 cells was lower than that of the sh-SNHG14-NC group: glycolysis and glycolytic capacity were decreased. Moreover, silencing SNHG14 decreased lactate production (Fig. 2d), glucose uptake (Fig. 2e), and expression of PKM2 and GLUT1 as compared to that of the sh-SNHG14-NC group (Fig. 2f). This was accompanied by a decrease in cell proliferation (Fig. 2g) and increase in apoptosis (Fig. 2h) in the sh-SNHG14 cells. Re-expressing SNHG14 in the sh-SNHG14 cells rescued the above phenotypes. Taken together, silencing SNHG14 suppressed glycolysis and proliferation, while promoting apoptosis in glioma cells.

### Lin28A facilitated aerobic glycolysis by stabilizing SNHG14

**Fig. 3:**
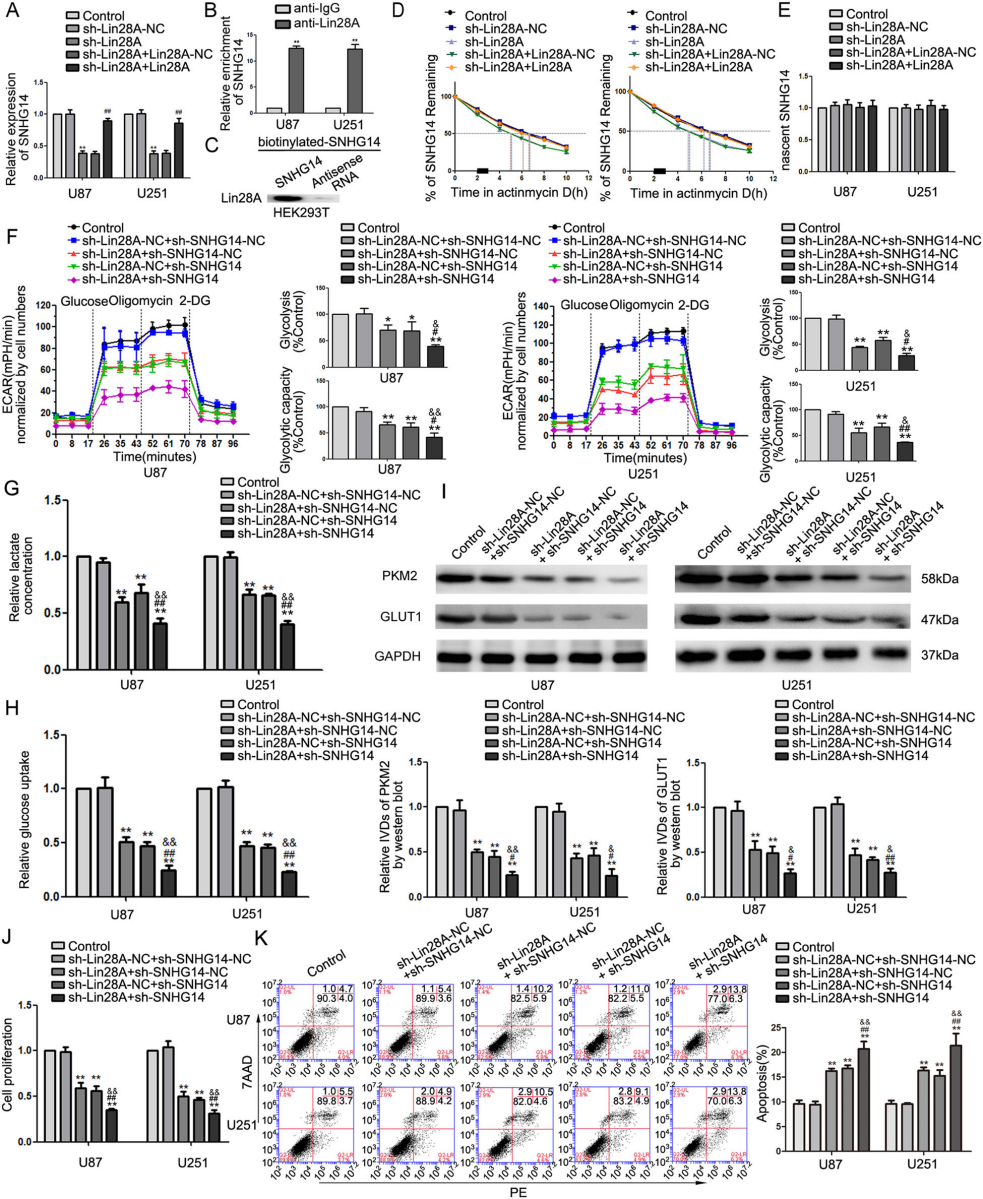
Lin28A facilitated aerobic glycolysis by stabilizing SNHG14. **a** SNHG14 was downregulated in the sh-Lin28A group, re-expression of Lin28A reversed SNHG14 expression. Data are presented as the mean ± SD (*n* = 3 in each group). **P < 0.01 versus sh-Lin28A-NC group (empty vector); ^##^*P* < 0.01 versus sh-Lin28A + Lin28A-NC group. **b** RNA immunoprecipitation (RIP) showed an enrichment of SNHG14 in Lin28A immunoprecipitated samples. Data are presented as the mean ± SD (*n* = 3, each group). **P < 0.01 versus anti-normal IgG group, using Student’s *t* test. **c** Immunoblotting for the specific associations of Lin28A with biotinylated-SNHG14 or antisense RNA from streptavidin RNA pulldown assay. **d** RNA half-life measurement to detect the *T*_1/2_ of SNHG14 upon Lin28A depletion or re-expression. **e** Click-iT Nascent RNA capture kit was conducted to label and capture newly synthesized RNA, and nascent SNHG14 was measured using qRT-PCR. **f** ECAR was measured to detect the effect of Lin28A and SNHG14 on glycolysis. **g**, **h** Lactate production and glucose uptake were measured upon depletion of Lin28A and SNHG14. **i** Expression of PKM2 and GLUT1 by western blot upon depletion of Lin28A and SNHG14. **j** CCK-8 assay was conducted to investigate the effect of Lin28A and SNHG14 on proliferation. **k** Flow cytometry analysis to evaluate the effect of depleting Lin28A and SNHG14 on apoptosis. Data are presented as the mean ± SD (*n* = 3 in each group). *P < 0.05, **P < 0.01 versus sh-Lin28A-NC + sh-SNHG14-NC group (empty vector); ^#^*P* < 0.05, ^##^*P* < 0.01 versus sh-Lin28A+sh-SNHG14-NC group; ^&^*P* < 0.05, ^&&^*P* < 0.01 versus sh-Lin28A-NC + sh-SNHG14 group. One-way analysis of variance was used for statistical analysis.

Since our results revealed the oncogenic nature of Lin28A and SNHG14 in glioma cells and the decrease in SNHG14 in the sh-Lin28A cells as compared to that in the sh-Lin28A-NC group (Fig. 3a), we speculated a positive correlation between SNHG14 and Lin28A. Using the StarBase database, Lin28A was predicted to bind SNHG14. As expected, RNA immunoprecipitation (RIP) showed an enrichment of SNHG14 in the Lin28A immunoprecipitated sample as compared to the pool in the IgG immunoprecipitated sample (Fig. 3b) and RNA pulldown assays demonstrated binding between SNHG14 and Lin28A (Fig. 3c). Fluorescence in situ hybridization (FISH) analysis also suggested that Lin28A and SNHG14 localized predominately in the cytoplasm (Supplementary Fig. S2g). Thus, Lin28A can bind SNHG14. RNA stability, which is measured by RNA half-life (*T*½), is crucial in RNA’s function; since actinomycin D blocks the de novo synthesis of RNA, we further used actinomycin D to determine the *T*½ of SNHG14; the *T*½ of SNHG14 reduced in the sh-Lin28A cells (Fig. 3d) as compared to that in sh-Lin28A-NC cells, while nascent SNHG14 showed no difference (Fig. 3e). We introduced a mutation in SNHG14 that interferes with its interaction with Lin28A. RIP showed reduced enrichment of mutant SNHG14 in the anti-Lin28A samples as compared to the wild-type SNHG14 group (Supplementary Fig. S3a). Silencing Lin28A in cells harboring wild-type SNHG14 reduced the *T*½ of SNHG14, cellular proliferation, lactate production, glucose uptake, glycolysis and glycolytic capacity as compared to that in sh-NC, while silencing Lin28A in SNHG14 mutant cells did not show the above phenotypes (Supplementary Fig. S3b–f). Subsequently, the glioma cells were cotransfected with sh-Lin28A and sh-SNHG14. As compared to the single knockdowns of Lin28A or SNHG14, double silencing further decreased glycolysis and glycolytic capacity, lactate production, glucose uptake, expression of PKM2 and GLUT1, and proliferation, while stimulating apoptosis (Fig. 3f–k). Thus, Lin28A facilitated aerobic glycolysis by stabilizing SNHG14, thereby enhancing cell proliferation and impairing apoptosis in glioma cells.

### IRF6 functions as a tumor suppressor and was downregulated in glioma cells and tissues

**Fig. 4:**
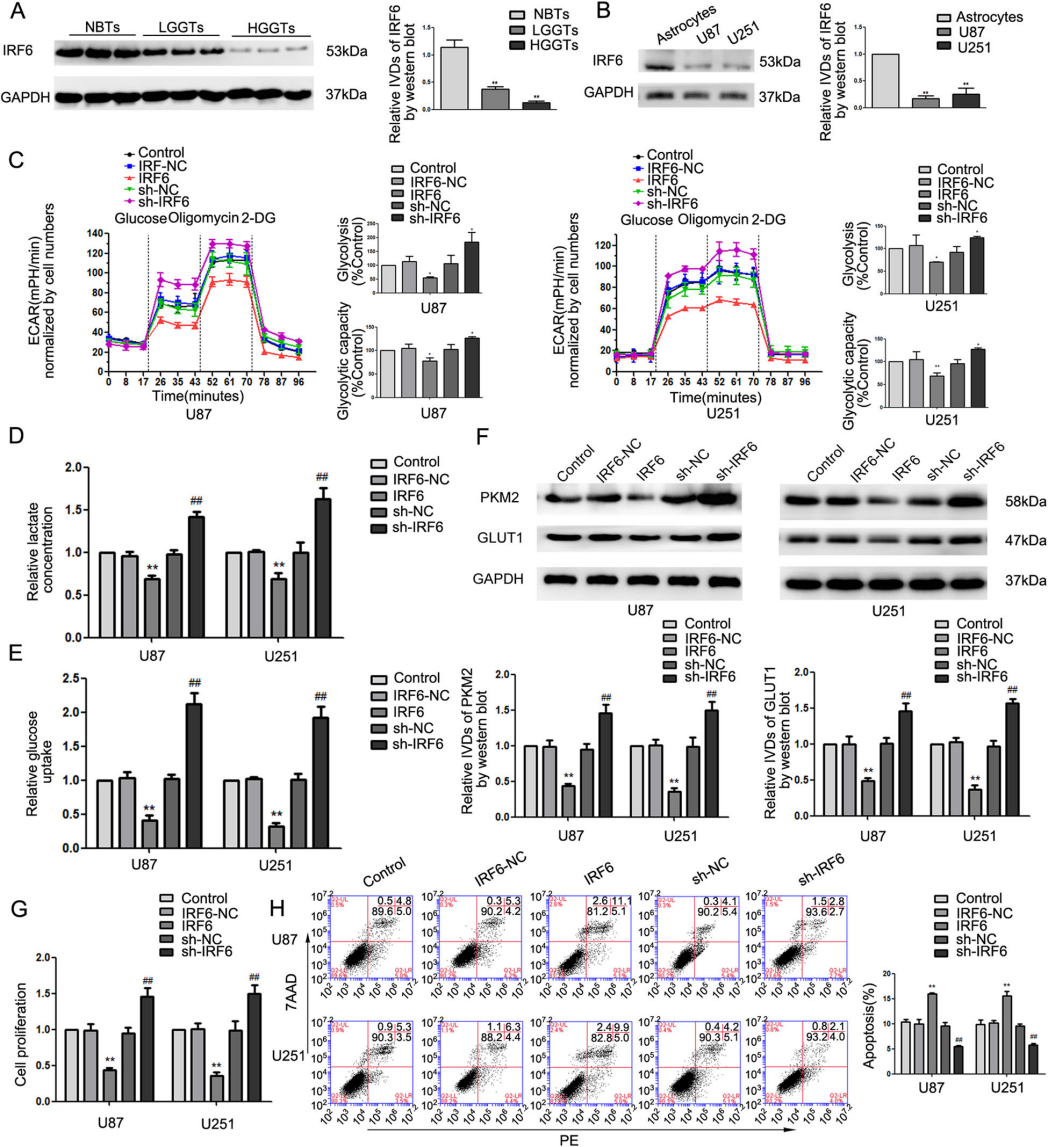
IRF6 functioned as a tumor suppressor and was downregulated in glioma cells and tissues. **a** Protein levels of IRF6 in NBTs and glioma tissues were measured by western blot. Data are presented as the mean ± SD (*n* = 3 in each group). **P < 0.01 versus NBTs group. **b** Protein levels of IRF6 in NHA, U87 and U251 cells. Data are presented as the mean ± SD (*n* = 3 in each group). **P < 0.01 versus NHA group. **c** ECAR was measured to detect the effect of IRF6 on glycolysis in U87 and U251 cells. **d**, **e** The lactate production and glucose uptake in response to overexpressing IRF6 or depletion. **f** Effect of IRF6 on the expression of PKM2 and GLUT1. **g** CCK-8 assay to investigate the effect of IRF6 on proliferation. **h** Flow cytometry analysis to evaluate the effect of IRF6 on apoptosis. Data are presented as the mean ± SD (*n* = 3 in each group). *P < 0.05, **P < 0.01 versus IRF6-NC group (empty vector); ^#^*P* < 0.05, ^##^*P* < 0.01 versus sh-IRF6-NC group (empty vector). One-way analysis of variance was used for statistical analysis.

The microarray showed an increase in IRF6 mRNA upon depleting SNHG14 (Supplementary Fig. S4a). The levels of IRF6 were lower in glioma tissues (as compared to NBTs; Fig. 4a), U87, and U251 cells (as compared to NHA; Fig. 4b). We generated stable IRF6-overexpressing/knockdown cell lines to investigate the role of IRF6 in glioma. Compared to the control group, overexpression of IRF6 inhibited glycolysis, decreased expression of PKM2, GLUT1 (Fig. 4c–f), and proliferation (Fig. 4g), while stimulating apoptosis in glioma cells (Fig. 4h). Notably, knockdown of IRF6 reversed these phenotypes (Fig. 4c–h). These results suggest that IRF6 impairs glycolysis, suppresses proliferation, and induces apoptosis in glioma cells.

### SNHG14 enhanced STAU1-mediated degradation of IRF6

**Fig. 5:**
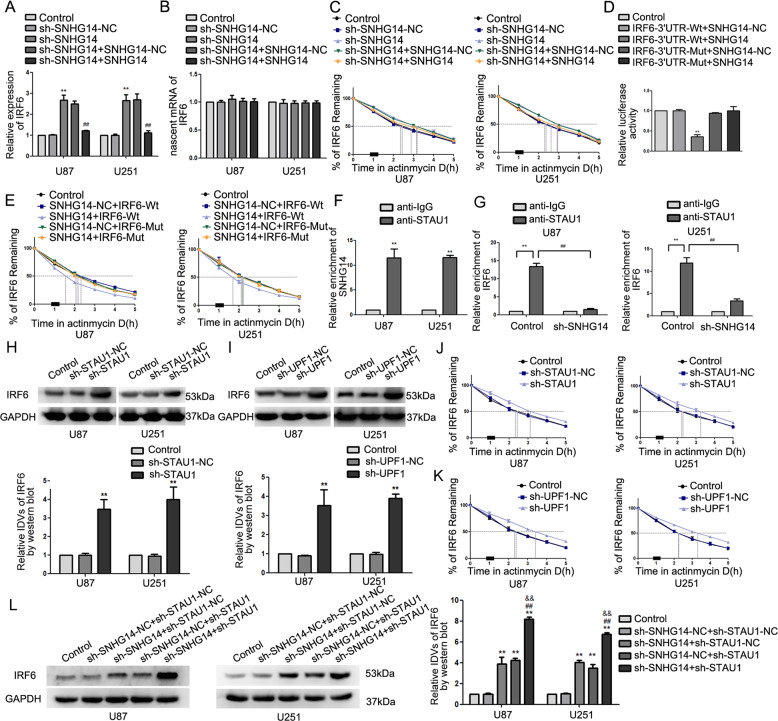
SNHG14 enhanced STAU1-mediated degradation of IRF6. **a** Expression of IRF6 upon depletion or re-expressing of SNHG14. Data are presented as mean ± SD (*n* = 3, each group). **P < 0.01 versus sh-SNHG14-NC group, ^##^*P* < 0.01 versus sh-SNHG14 + SNHG14-NC group. **b** Nascent SNHG14 was measured using qRT-PCR. **c** *T*_1/2_ of IRF6 mRNA was detected upon SNHG14 depletion or re-expression. **d** Relative luciferase activity was conducted in wild-type or mutant IRF6 3′UTR cells. Data are presented as the mean ± SD (*n* = 3, each group). **e** *T*_1/2_ of IRF6 mRNA was detected with overexpressing SNHG14 in wild-type or mutant IRF6 3′UTR cells. **f** Relative enrichment of SNHG14 in STAU1 immunoprecipitation group by RIP assay. Data are represented as mean ± SD (*n* = 3, each group). **P < 0.01 versus anti-IgG group. **g** The enrichment of IRF6 in the STAU1 immunoprecipitation group. Data are represented as mean ± SD (*n* = 3, each group). **P < 0.01 versus anti-IgG group; ^##^*P* < 0.01 versus control group. **h** Effect of STAU1 on IRF6 expression. Data are presented as the mean ± SD (*n* = 3, each group). **P < 0.01 vs. sh-STAU1-NC group. **i** Effect of UPF1 on IRF6 expression. Data are presented as the mean ± SD (*n* = 3, each group). **P < 0.01 vs. sh-UPF1-NC group. **j** *T*_1/2_ of IRF6 mRNA regulated by STAU1. Data are presented as the mean ± SD (*n* = 3, each group). **P < 0.01 versus sh-STAU1-NC. **k** *T*_1/2_ of IRF6 mRNA regulated by UPF1. Data are presented as the mean ± SD (*n* = 3, each group). **P < 0.01 versus sh-UPF1-NC. **l** Effect of depleting SNHG14 and STAU1 on IRF6 expression. Data are presented as the mean ± SD (*n* = 3, each group). **P < 0.01 versus sh-SNHG14-NC + sh-STAU1-NC; ^##^*P* < 0.01 versus sh-SNHG14 + sh-STAU1-NC group; ^&&^*P* < 0.01 versus sh-SNHG14-NC + sh-STAU1 group. One-way analysis of variance was used for statistical analysis.

RNA (Fig. 5a) and protein levels (Supplementary Fig. S4b) of IRF6 significantly increased in response to SNHG14 depletion. Nascent IRF6 mRNA levels were unchanged (Fig. 5b), but the *T*½ of IRF6 mRNA increased in sh-SNHG14 cells (Fig. 5c); re-expressing SNHG14 reversed the above changes. Using the IntaRNA database, we determined that IRF6 possesses a specific sequence that can be targeted by SNHG14. Thus, we mutated this sequence in the 3′ untranslated region (3′-UTR) of IRF6 (Supplementary Fig. S4c). Dual-luciferase reporter assay confirmed this predicted binding site (Fig. 5d). Overexpressing SNHG14 in wild-type IRF6-expressing cells decreased the *T*½ of IRF6 mRNA (Fig. 5e) and enhanced the cellular proliferation and glycolytic flux as compared to that in sh-NC, while overexpressing SNHG14 in mutant IRF6-expressing cells reverses the above phenotypes (Supplementary Fig. S4d–g). These results suggested that SNHG14 might affect IRF6 mRNA degradation in the specific sequence manner. SMD (STAU1-mediated degradation) is a very common RNA degradation pathway in mammals. STAU1 recognizes the STAU1 binding site (SBS) in the 3′ UTR of the target mRNA and recruits UPF1 to promote mRNA degradation. RIP was used to detect the interaction between SNHG14/IRF6 and STAU1. SNHG14 were enriched in the STAU1 immunoprecipitated sample as compared to the anti-IgG group (Fig. 5f). IRF6 mRNA were enriched in the STAU1 immunoprecipitated sample as compared to the anti-IgG group; depleting SNHG14 significantly reduced the enrichment of IRF6 in the STAU1 immunoprecipitated samples (Fig. 5g).

To determine whether STAU1 and UPF1 mediated the interaction between SNHG14 and IRF6 mRNA, we depleted STAU1 or UPF1. IRF6 was upregulated in cells depleted of STAU1 or UPF1 as compared to the control cells (Fig. 5h, i, Supplementary Fig. S4h, i). Moreover, the *T*½ of IRF6 mRNA increased upon depleting STAU1 (Fig. 5j) or UPF1 (Fig. 5k) compared to that in controls; depleting SNHG14 and STAU1 further increased the expressions of IRF6 (Fig. 5l). These results demonstrated reciprocal repression between SNHG14 and IRF6; knockdown of SNHG14 blocked SMD pathway, thereby resulting in increased mRNA levels of IRF6, suppressing glycolysis and proliferation in glioma.

### SNHG14 promoted the expression of PKM2 and GLUT1 and accelerated aerobic glycolysis by downregulating IRF6

**Fig. 6:**
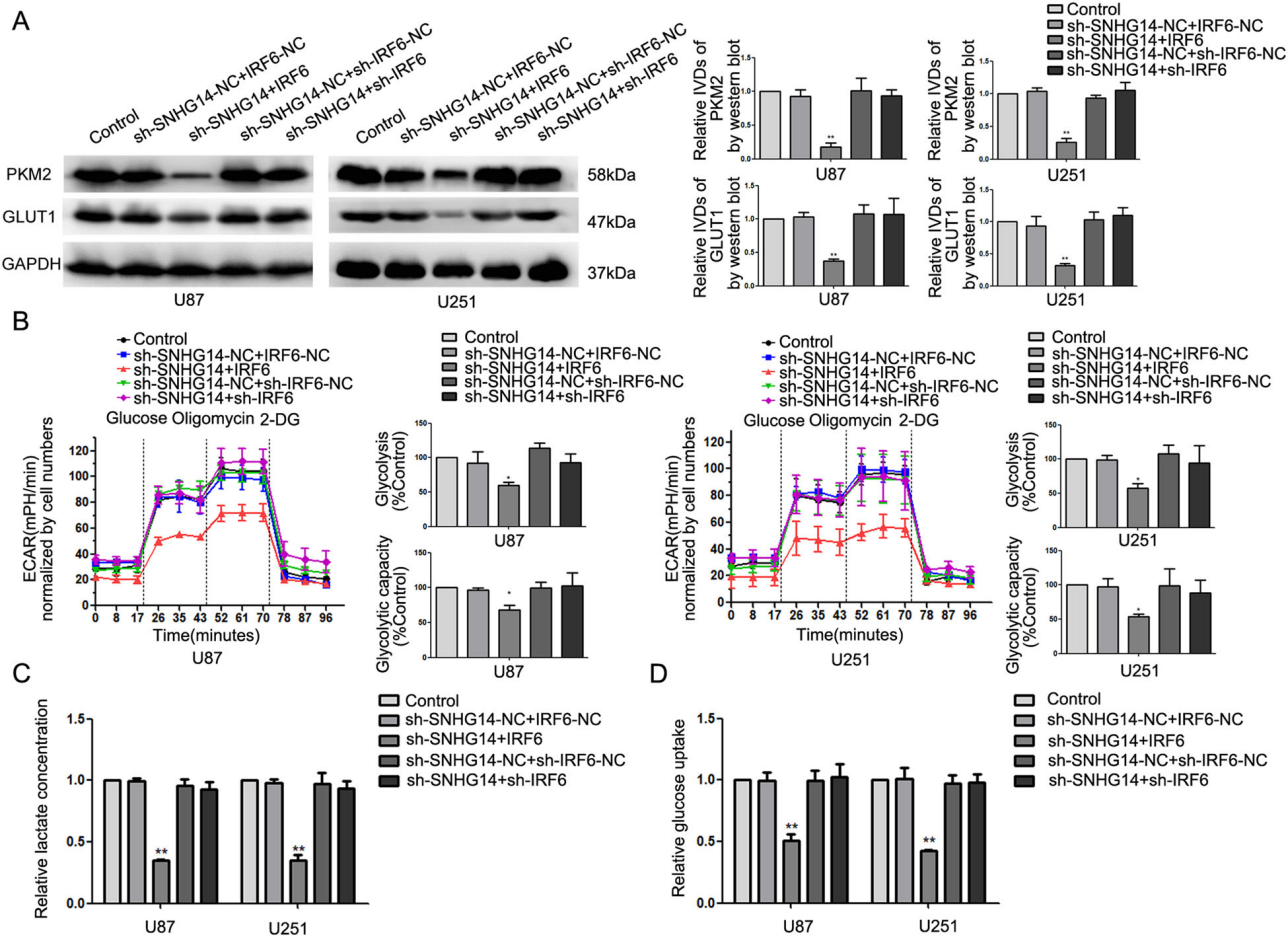
SNHG14 promoted the expression of PKM2 and GLUT1 and accelerated aerobic glycolysis by downregulating IRF6. **a** Expression of PKM2 and GLUT1 upon the altered expression of SNHG14 and IRF6. **b** ECAR was measured to detect the effect on glycolysis upon the altered expression of SNHG14 and IRF6. **c**, **d** Lactate production and glucose uptake were measured to detect the effect on glycolysis with the altered expression of SNHG14 and IRF6. Data are presented as the mean ± SD (*n* = 3, each group). *P < 0.05, **P < 0.01 versus sh-SNHG14-NC + IRF6-NC group. One-way analysis of variance was used for statistical analysis.

After the mechanism of IRF6 regulation was studied, we further explored the phenotype upon changing of expression of SNHG14 and IRF6. Phenotypes induced by depleting SNHG14 (decreased the expression of PKM2 and GLUT1; inhibition of glycolysis and glycolytic capacity, lactate production and glucose uptake; suppression of proliferation; enhanced apoptosis) could be reversed by silencing IRF6 in glioma cells (Fig. 6a–d, Supplementary Fig. S4j, k). Moreover, simultaneously depleting SNHG14 and overexpressing IRF6 advanced those phenotypes caused by depleting SNHG14 (Fig. 6a–d, Supplementary Fig. S4j, k).

### IRF6 inhibited the expression of PKM2 and GLUT1 by targeting their promoters

**Fig. 7:**
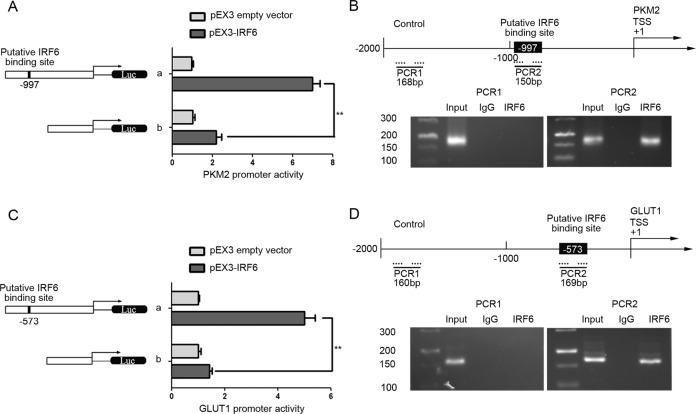
IRF6 inhibited the expression of PKM2 and GLUT1 by targeting their promoters. **a**, **c** Schematic depiction of the different reporter constructs used and the luciferase activity of PKM2 (**a**) and GLUT1 (**c**) are shown. The *Y*-bar shows the position of the deletions on the DNA fragments. *X*-bar shows the promoter activity of constructed plasmid after normalization with the cotransfected reference vector, and expressed as relative to the activity of pEX3 empty vector, which the activity was set to 1. Data are represented as the mean ± SD (*n* = 3, each). **P < 0.01 vs. b + pEX3-IRF6 group. **b** Schematic representation of PKM2 promoter region. Chromatin immunoprecipitation to show the products for amplified putative IRF6-binding sites of PKM2. **d** Schematic representation of GLUT1 promoter region. Chromatin immunoprecipitation to show the products for amplified putative IRF6-binding sites of GLUT1. Images are representative of three independent experiments.

Since overexpressing IRF6 significantly decreased the expression of PKM2 and GLUT1, we further determined the underlying molecular mechanism(s). The transcription start site (TSS) for PKM2 and GLUT1 was predicted by DBTSS Home. A putative IRF6-binding sites with the promoter regions in PKM2 was predicted by the JASPAR database. A version of IRF6 without the predicted binding region was generated to perform luciferase assays. Compared to the full-length PKM2 promoter, deleting the −997 site (b) significantly reduced the PKM2 promoter activity (Fig. 7a). To explore whether IRF6 directly associated with the promoter of PKM2, chromatin immunoprecipitation was applied. There were 150 bp-long amplicons at the predicted binding sites, but none in negative control region (Fig. 7b), suggesting that IRF6 specifically binds to the putative binding site of PKM2.

Similarly, a putative IRF6-binding site with the promoter regions in GLUT1 was predicted; deleting the −573 site (b) significantly reduced GLUT1 promoter activity (Fig. 7c). Chromatin immunoprecipitation revealed that there were 169 bp-long amplicons at the predicted binding sites (Fig. 7d), suggesting that IRF6 specifically binds to the putative binding site of GLUT1.

### Simultaneous depletion of Lin28A and SNHG14 with IRF6 overexpression restrained tumor growth and prolonged survival in nude mice

**Fig. 8:**
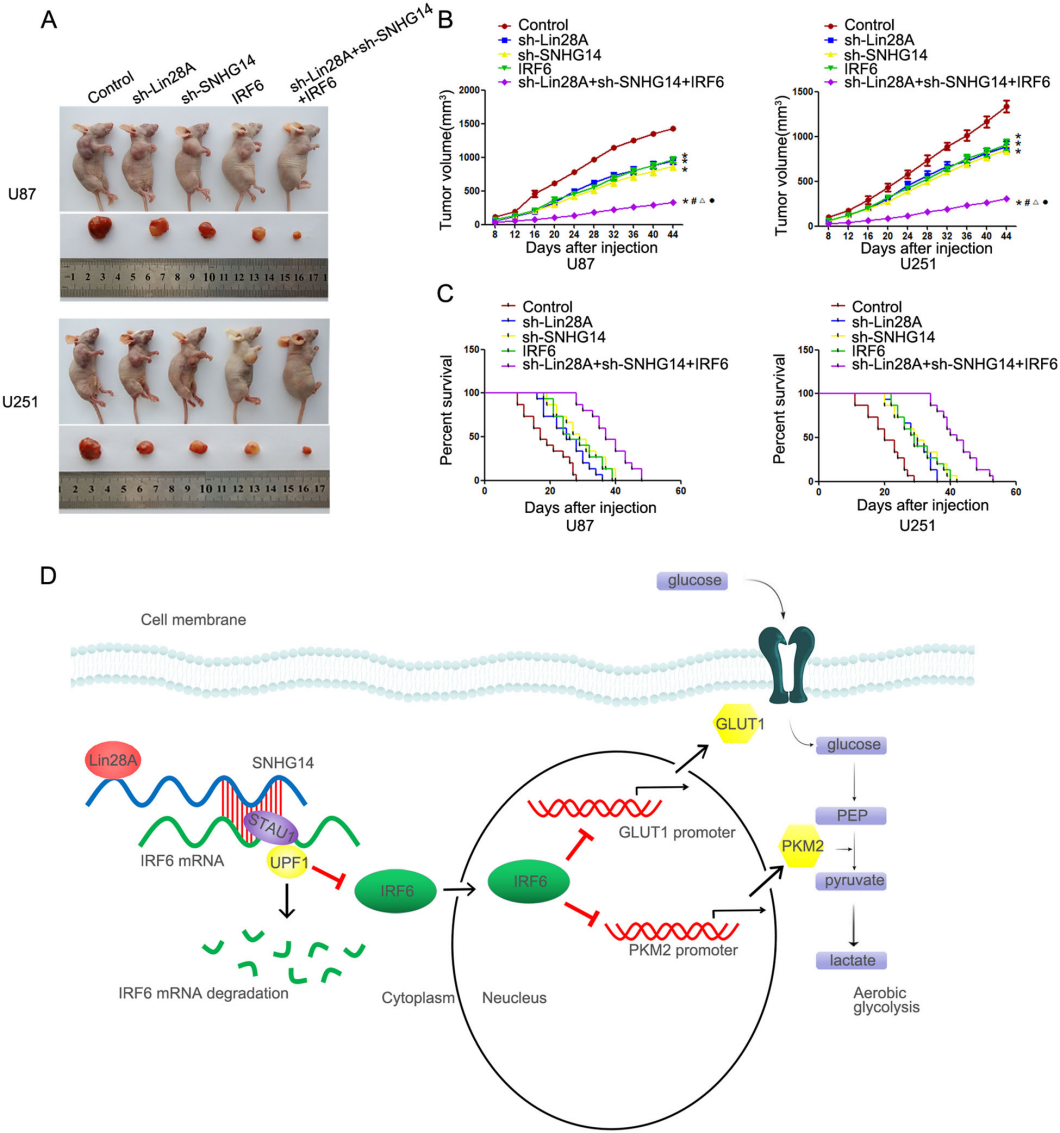
Depletion of Lin28A and SNHG14 with IRF6 overexpression restrained tumor growth and prolonged survival in nude mice. **a** The nude mice carrying tumors and the sample tumors from respective group are shown. **b** Tumors excised from the nude mice in different groups after 44 days. *P < 0.05, versus sh-Lin28A-NC + sh-SNHG14-NC + IRF6-NC group; ^#^*P* < 0.05 versus sh-Lin28A group; ^△^*P* < 0.05 versus sh-SNHG14 group. ^•^*P* < 0.05 versus IRF6 group. One-way analysis of variance was used for statistical analysis. **c** The survival curves of nude mice with orthotopic xenografts. *P* < 0.05 for sh-Lin28A group or sh-SNHG14 group or IRF6 group versus sh-Lin28A-NC + sh-SNHG14-NC + IRF6-NC group; *P* < 0.05 for sh-Lin28A+sh-SNHG14 + IRF6 group versus sh-Lin28A-NC + sh-SNHG14-NC + IRF6-NC group. Log-rank test was used for statistical analysis. **d** The schematic cartoon of the mechanism of Lin28A/SNHG14/IRF6 axis, functions as a potential aerobic glycolysis enhancer in glioma.

Subcutaneous and orthotopic xenografts were used to confirm our results from glioma cells and tissues. The nude mice carrying tumors and the sample tumors from respective group are shown in Fig. 8a. The average independent size of the xenograft was small in Lin28A-depleted, SNHG14-depleted, and IRF6-overexpressing mice as compared to that in the controls (Fig. 8b). Among the others, cells depleted of Lin28A and SNHG14 and overexpressing IRF6 had the smallest tumor volumes (Fig. 8b). Consistent with the results using the subcutaneous xenograft, mice depleted of Lin28A or SNHG14 or overexpressing IRF6 transplanted with the orthotopic xenograft exhibited longer survival as compared to that of controls; the combination of them exhibited the longest survival (Fig. 8c). The schematic cartoon of the mechanism of Lin28A/SNHG14/IRF6 axis functions as a potential aerobic glycolysis enhancer in glioma (Fig. 8d).

Notably, as compared to depleting Lin28A, depleting SNHG14, or overexpressing IRF6, glycolysis and glycolytic capacity, as well as proliferation were increased in the PKM2 overexpression group (Supplementary Fig. S5a–f).

## Discussion

This study reveals the oncogenic nature of Lin28A and SNHG14, while IRF6 functions as a tumor suppressor in glioma. Depletion of Lin28A destabilized and decreased SNHG14, downregulated SNHG14 increased IRF6 mRNA levels by blocking its degradation via SMD pathway. IRF6 bound the promoters of PKM2 and GLUT1, and inhibited their transcription expression, thereby impairing aerobic glycolysis and tumorigenesis in glioma. To the best of our knowledge, this study is the first to demonstrate the role of Lin28A/SNHG14/IRF6 axis in regulating aerobic glycolysis in glioma cells.

RBPs are crucial in glycometabolism. Lin28A is overexpressed and promotes the Warburg effect in glioma cells. Consistent with our results, the Oncomine database shows Lin28A is overexpressed in glioblastoma that attributes to shorter survival. Lin28A is abundant in liver cancer and stimulates aerobic glycolysis and cancer progression by upregulating 3-phosphoinositide-dependent kinase-1 (PDK1)^10^. In human embryonic kidney cells, Lin28A modulates glycolysis by upregulating hexokinase II^25^.

SNHG14 stimulates migration and invasiveness in clear cell renal cell carcinoma^12^, breast cancer^13^, and gastric cancer^14^. Our results showed that SNHG14 was overexpressed and promoted the Warburg effect in glioma, suggesting that SNHG14 regulates glycometabolism in glioma. Consistent with our results, the lncRNA UCA1 promotes the expression of fructose-2,6-diphosphatase and facilitates aerobic glycolysis in glioma^26^. Depleting lncRNA LINK-A in glioma significantly reduces the expression of LDH-A and inhibits aerobic glycolysis^27^.

Among multiple regulatory roles, RBPs can bind to lncRNAs and regulate their stability, thereby performing diverse biological functions. HuR promotes malignancy by stabilizing the lncRNA NEAT1 in ovarian cancer^28^. PABPC1 promotes proliferation and migration in bladder cancer by stabilizing the lncRNA PAGBC^29^. Downregulation of Lin28A destabilizes MALAT1 and prevents malignant transformation in osteoma^30^. LncRNA microarrays showed that SNHG14 was significantly downregulated in Lin28A-depleted glioma cells. Therefore, we speculated that Lin28A might regulate SNHG14 in glioma cells. As shown by FISH, RIP, and RNA pulldown assays, Lin28A bound SNHG14. Further, we generated an SNHG14 mutant that interfered with the interaction between Lin28A and SNHG14. Phenotypes induced by depleting Lin28A were reversed in cells expressing wild-type SNHG14, but not in SNHG14 mutant cells. Our findings demonstrated that the downregulation of Lin28A destabilized SNHG14, thereby reducing its expression and inhibiting glycolysis and proliferation.

IRF6 regulates innate immunity, cell cycle arrest, and biological behavior of tumors^31–33^. According to our microarray results, IRF6 was significantly upregulated in SNHG14-depleted cells. This indicates that IRF6 might be a downstream target of SNHG14. IRF6 was downregulated in glioma; overexpressing IRF6 significantly reduced aerobic glycolysis and cell proliferation, and promoted apoptosis. This suggests that IRF6 acts as a tumor suppressor in glioma. Studies have reported low levels of IRF6 in epithelial tissues and also its role in suppressing cancer growth^34^, such as nasopharyngeal^35^, breast^36^, esophageal^16^, and vulvar cancers^32^. Glioma cells originate from neuroepithelial layers. To the best of our knowledge, this is the first report on the reduced expression of IRF6 in glioma cells; overexpressing IRF6 inhibited glycolysis, and suppressed cell proliferation.

SMD is very common in mammals. STAU1 recognizes the STAU1 binding site in the 3′ UTR of the target mRNA and recruits UPF1 that degrades the mRNA^37,38^. LncRNAs promote the degradation of RNA via the SMD pathway. SNHG5 promotes colorectal cancer by stabilizing its target SPATS2 via inhibiting SMD^39^. We identified a potential SBS sequence in the 3′ UTR of IRF6 and generated a mutation. Mutant of IRF6 prevented SNHG14 from regulating the *T*½ of IRF6 mRNA. Further, depleting SNHG14 significantly reduced the binding of IRF6 with STAU1. Downregulating STAU1 or UPF1 prevented the degradation of IRF6 mRNA. Taken together, our findings demonstrate that SBS, consisting of SNHG14, STAU1 and specific sequence in the 3′ UTR of IRF6, is crucial in SNHG14-regulated degradation of IRF6 mRNA; SNHG14 promotes the degradation of IRF6 mRNA via the SMD pathway, thereby enhancing aerobic glycolysis and cell proliferation in glioma.

PKM2 is a key enzyme in aerobic glycolysis in cancer cells^21^. PKM2 is upregulated and promotes cell proliferation, Warburg effect, and tumorigenesis in glioma^40,41^. GLUT1 is the primary GLUT that facilitates glucose uptake in brain. Overexpression of IRF6 significantly decreased the expression of PKM2 and GLUT1 by targeting 3′UTR of their promoter, and reduces their transcription. Similar to our results, it has been reported that IRF6 directly binds the promoter of ABCG2, reduces its transcription, and decreases ABCG2 expression, thereby inhibiting proliferation and reversing the phenotype of nasopharyngeal carcinoma stem cells^42^.

In summary, the study provides novel insights into the role and function of the Lin28A/SNHG14/IRF6 axis in regulating glycometabolism in human glioma. Single or combination therapy targeting Lin28A, SNHG14, and IRF6 has the potential in clinical settings and may help develop new therapeutic strategies for treating glioma.

## Supplementary information

Supplementary table 1

Supplymentary figure 1

Supplymentary figure 2

Supplymentary figure 3

Supplymentary figure 4

Supplymentary figure 5

Supplymentary figure legends

## References

[1] Thorne AH (2014). Role of cysteine-rich 61 protein (CCN1) in macrophage-mediated oncolytic herpes simplex virus clearance. Mol. Ther..

[2] Hanahan D, Weinberg RA (2011). Hallmarks of cancer: the next generation. Cell.

[3] Warburg O (1956). On the origin of cancer cells. Science.

[4] DeBerardinis RJ (2007). Beyond aerobic glycolysis: transformed cells can engage in glutamine metabolism that exceeds the requirement for protein and nucleotide synthesis. Proc. Natl. Acad. Sci. USA.

[5] Parsons DW (2008). An integrated genomic analysis of human glioblastoma multiforme. Science.

[6] Wolf A (2011). Hexokinase 2 is a key mediator of aerobic glycolysis and promotes tumor growth in human glioblastoma multiforme. J. Exp. Med..

[7] West JA (2009). A role for Lin28 in primordial germ-cell development and germ-cell malignancy. Nature.

[8] Fang T (2017). Musashi 2 contributes to the stemness and chemoresistance of liver cancer stem cells via LIN28A activation. Cancer Lett..

[9] He, F. et al. Long noncoding RNA PVT1-214 promotes proliferation and invasion of colorectal cancer by stabilizing Lin28 and interacting with miR-128. *Oncogene* **38**, 164 (2018). 10.1038/s41388-018-0432-8 PMC6329639 30076414

[10] Ma X (2014). Lin28/let-7 axis regulates aerobic glycolysis and cancer progression via PDK1. Nat. Commun..

[11] Dong Z (2017). Inhibition of neurotensin receptor 1 induces intrinsic apoptosis via let-7a-3p/Bcl-w axis in glioblastoma. Br. J. Cancer.

[12] Liu G, Ye Z, Zhao X, Ji Z (2017). SP1-induced up-regulation of lncRNA SNHG14 as a ceRNA promotes migration and invasion of clear cell renal cell carcinoma by regulating N-WASP. Am. J. Cancer Res..

[13] Dong H (2018). Long non-coding RNA SNHG14 induces trastuzumab resistance of breast cancer via regulating PABPC1 expression through H3K27 acetylation. J. Cell Mol. Med..

[14] Liu Z, Yan Y, Cao S, Chen Y (2018). Long non-coding RNA SNHG14 contributes to gastric cancer development through targeting miR-145/SOX9 axis. J. Cell Biochem..

[15] Qi X (2017). Long non-coding RNA SNHG14 promotes microglia activation by regulating miR-145-5p/PLA2G4A in cerebral infarction. Neuroscience.

[16] Botti E (2011). Developmental factor IRF6 exhibits tumor suppressor activity in squamous cell carcinomas. Proc. Natl. Acad. Sci. USA.

[17] Slattery ML, Lundgreen A, Bondurant KL, Wolff RK (2011). Interferon-signaling pathway: associations with colon and rectal cancer risk and subsequent survival. Carcinogenesis.

[18] Kim KY (2015). Expression analyses revealed thymic stromal co-transporter/Slc46A2 is in stem cell populations and is a putative tumor suppressor. Mol. Cells.

[19] Lin Y (2016). Upregulation of interferon regulatory factor 6 promotes neuronal apoptosis after traumatic brain injury in adult rats. Cell Mol. Neurobiol..

[20] Guo H (2016). miRNA-451 inhibits glioma cell proliferation and invasion by downregulating glucose transporter 1. Tumour Biol..

[21] Mazurek S (2011). Pyruvate kinase type M2: a key regulator of the metabolic budget system in tumor cells. Int. J. Biochem. Cell Biol..

[22] Christofk HR (2008). The M2 splice isoform of pyruvate kinase is important for cancer metabolism and tumour growth. Nature.

[23] Sun Q (2011). Mammalian target of rapamycin up-regulation of pyruvate kinase isoenzyme type M2 is critical for aerobic glycolysis and tumor growth. Proc. Natl. Acad. Sci. USA.

[24] Yang W (2012). EGFR-Induced and PKCε monoubiquitylation-dependent NF-κB activation upregulates PKM2 expression and promotes tumorigenesis. Mol. Cell.

[25] Docherty CK, Salt IP, Mercer JR (2016). Lin28A induces energetic switching to glycolytic metabolism in human embryonic kidney cells. Stem Cell Res. Ther..

[26] He Z, You C, Zhao D (2018). Long non-coding RNA UCA1/miR-182/PFKFB2 axis modulates glioblastoma-associated stromal cells-mediated glycolysis and invasion of glioma cells. Biochem. Biophys. Res. Commun..

[27] Wu D, Zhao B, Cao X, Wan J (2017). Long non-coding RNA LINK-A promotes glioma cell growth and invasion via lactate dehydrogenase A. Oncol. Rep..

[28] Chai Y, Liu J, Zhang Z, Liu L (2016). HuR-regulated lncRNA NEAT1 stability in tumorigenesis and progression of ovarian cancer. Cancer Med..

[29] Wu XS (2017). LncRNA-PAGBC acts as a microRNA sponge and promotes gallbladder tumorigenesis. EMBO Rep..

[30] Wang Z (2018). RNA binding protein Lin28A promotes osteocarcinoma cells progression by associating with the long noncoding RNA MALAT1. Biotechnol. Lett..

[31] Ferretti E (2011). A conserved Pbx-Wnt-p63-Irf6 regulatory module controls face morphogenesis by promoting epithelial apoptosis. Dev. Cell.

[32] Rotondo JC (2016). Hypermethylation-induced inactivation of the IRF6 gene as a possible early event in progression of vulvar squamous cell carcinoma associated with lichen sclerosus. JAMA Dermatol..

[33] Bailey CM, Abbott DE, Margaryan NV, Khalkhali-Ellis Z, Hendrix MJ (2008). Interferon regulatory factor 6 promotes cell cycle arrest and is regulated by the proteasome in a cell cycle-dependent manner. Mol. Cell Biol..

[34] Bailey CM, Hendrix MJ (2008). IRF6 in development and disease: a mediator of quiescence and differentiation. Cell Cycle.

[35] Xu, L. et al. The developmental transcription factor IRF6 attenuates ABCG2 gene expression and distinctively reverses stemness phenotype in nasopharyngeal carcinoma. *Cancer Lett.* **432**, 230 (2017). 10.1016/j.canlet.2017.10.016 29111349

[36] Bailey CM (2005). Mammary serine protease inhibitor (Maspin) binds directly to interferon regulatory factor 6: identification of a novel serpin partnership. J. Biol. Chem..

[37] Kim YK, Furic L, Desgroseillers L, Maquat LE (2005). Mammalian Staufen1 recruits Upf1 to specific mRNA 3’UTRs so as to elicit mRNA decay. Cell.

[38] Kim MY (2014). Staufen1-mediated mRNA decay induces Requiem mRNA decay through binding of Staufen1 to the Requiem 3’UTR. Nucleic Acids Res..

[39] Damas ND (2016). SNHG5 promotes colorectal cancer cell survival by counteracting STAU1-mediated mRNA destabilization. Nat. Commun..

[40] Yang W (2012). ERK1/2-dependent phosphorylation and nuclear translocation of PKM2 promotes the Warburg effect. Nat. Cell Biol..

[41] Yang W (2011). Nuclear PKM2 regulates beta-catenin transactivation upon EGFR activation. Nature.

[42] Xu L (2018). The developmental transcription factor IRF6 attenuates ABCG2 gene expression and distinctively reverses stemness phenotype in nasopharyngeal carcinoma. Cancer Lett..

