# Supplementary material for: Lin28A promotes IRF6-regulated aerobic glycolysis in glioma cells by stabilizing SNHG14
Source: Cell Death Dis. 2020 Jun 11;11(6):447. doi: 10.1038/s41419-020-2650-6 (PMC7289837; doi:10.1038/s41419-020-2650-6)
Supplement: Supplementary file 1 — Supplementary table 1 [file 41419_2020_2650_MOESM1_ESM.doc]

| Primer | Gene | Sequence (5'->3') or Assay ID |
| --- | --- | --- |
| Primer | Lin28A | F:CATGCAGAAGCGCAGATCA |
|  |  | R:AGCGGACATGAGGCTACCAT |
|  | SNHG14 | F:TGAGCGGCATAGATGGTGAC  R:AGCTCAGTGCAGCAGACCAG |
|  | IRF6 | F:ATGATGGCACCAAGGAGGTG  R:CTGGATGGGAACATGGTGCT |
|  | GAPDH | F:AAATCCCATCACCATCTTCCAG |
|  |  | R:TGATGACCCTTTTGGCTCCC |

Table S1. Primers used for RT-qPCR.

One-Step SYBR PrimeScript RT-PCR cycling conditions were as follows: 5 minutes at 42°C, 10 seconds at 95°C, 40 cycles of 3 seconds at 95°C, and 30 seconds at 60°C.

Table S2. Primary antibodies information used in the Western blotting

| Antibody | Host | Manufacturer | Concertration |
| --- | --- | --- | --- |
| Lin28A | Rabbit | Proteintech,Rosemont, IL | 1:500 |
| IRF6 | Rabbit | Proteintech,Rosemont, IL | 1:700 |
| PKM2 | mouse | Proteintech,Rosemont, IL | 1:1500 |
| GLUT1 | mouse | Proteintech,Rosemont, IL | 1:800 |
| GAPDH | mouse | Proteintech,Rosemont, IL | 1:1000 |

Table S3. Target Sequences of shRNA

| Gene |  | Target Sequence(5'->3') |
| --- | --- | --- |
| SNHG14 | SNHG14-RNAi(64271-1) | TGGGAGCTAAATAGTTCAGAA |
|  | SNHG14-RNAi(64272-2) | TTGCTTTAAACCCACCACTTA |
|  | SNHG14-RNAi(64273-1) | TTGCTGATATTTAAGGCACTA |
| IRF6 | IRF6-homo-388 | GCTACACAGGGACTCTAAACG |
|  | IRF6-homo-1449 | GGAAACCATTGGAAAGGAAAC |
| LIN28A | LIN28A -homo-449 | CTGGTGGAGTATTCTGTATTG |
|  | LIN28A -homo-388 | GCAGTGGAGTTCACCTTTAAG |
|  | LIN28A -homo-239 | GCATCTGTAAGTGGTTCAACG |

Table S4. Primers used for ChIP experiments

| Gene | Binding site or Control | Sequence (5'->3') | Product size (bp) | Annealing temperature (°C) |
| --- | --- | --- | --- | --- |
| PKM2 | PCR1 | F: CTCCCGAATAGCTGGGACTA | 168 | 57.7 |
|  |  | R: ATTGGCTCACGCCTGTAATC |  |  |
|  | PCR2 | F: CTCTCGGATTCAAGCGATTC | 150 | 58.4 |
|  |  | R: AATCACATGAGGCCAGGAGTT |  |  |
| GLUT1 | PCR1 | F: TGTCCTCAAGGTGCTCACAG | 160 | 58.2 |
|  |  | R: GACCTCACCATGCCAAAAAG |  |  |
|  | PCR2 | F: GAGCGAGACTCTGTCTGAGAAAA | 169 | 57.6 |
|  |  | R: GGCAGGCTAACTGACTGACA |  |  |
