# Supplementary figures and images for: Lin28A promotes IRF6-regulated aerobic glycolysis in glioma cells by stabilizing SNHG14
Source: Cell Death Dis. 2020 Jun 11;11(6):447. doi: 10.1038/s41419-020-2650-6 (PMC7289837; doi:10.1038/s41419-020-2650-6)

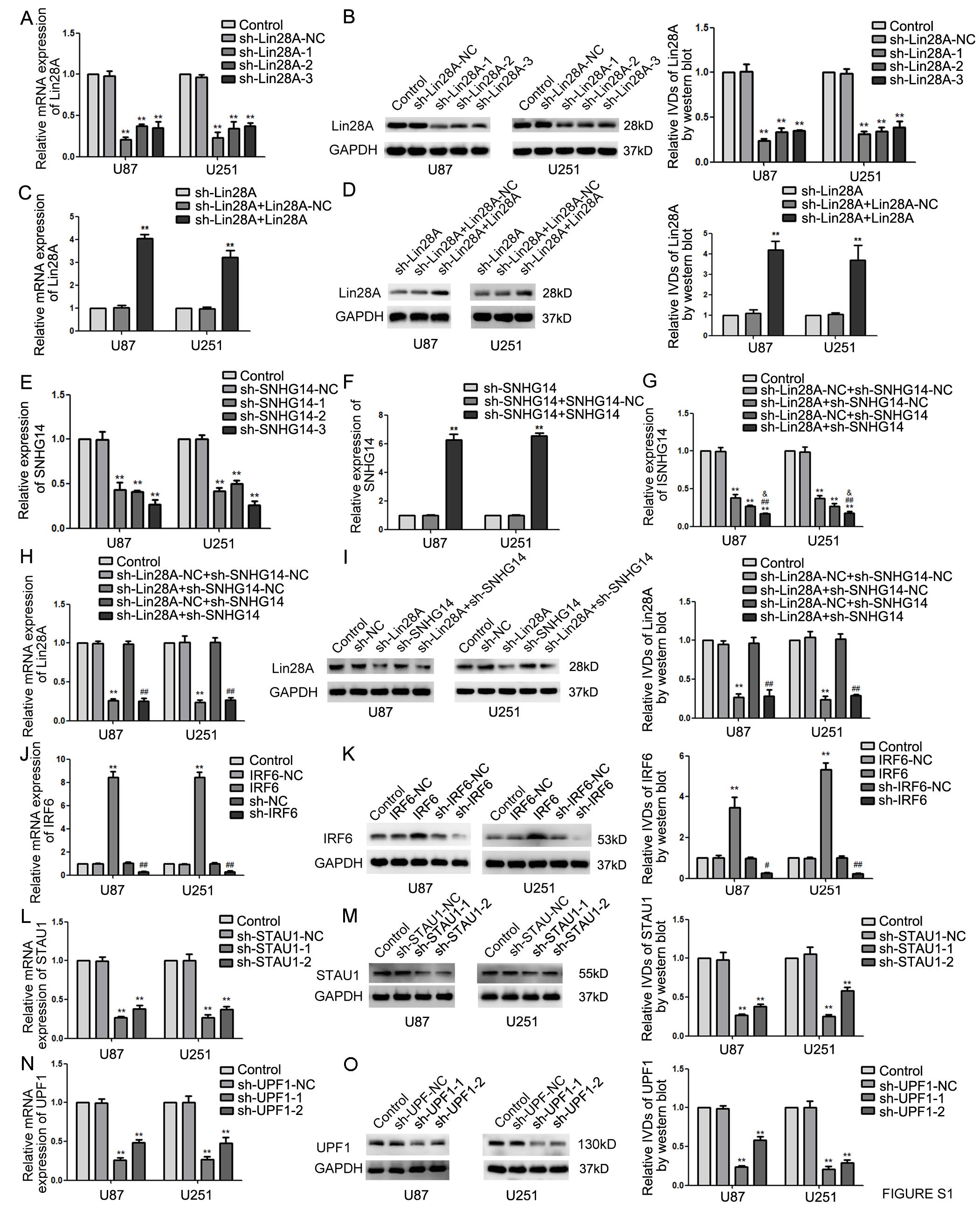

Supplement: Supplementary file 2 — Supplymentary figure 1 [file 41419_2020_2650_MOESM2_ESM.tif]

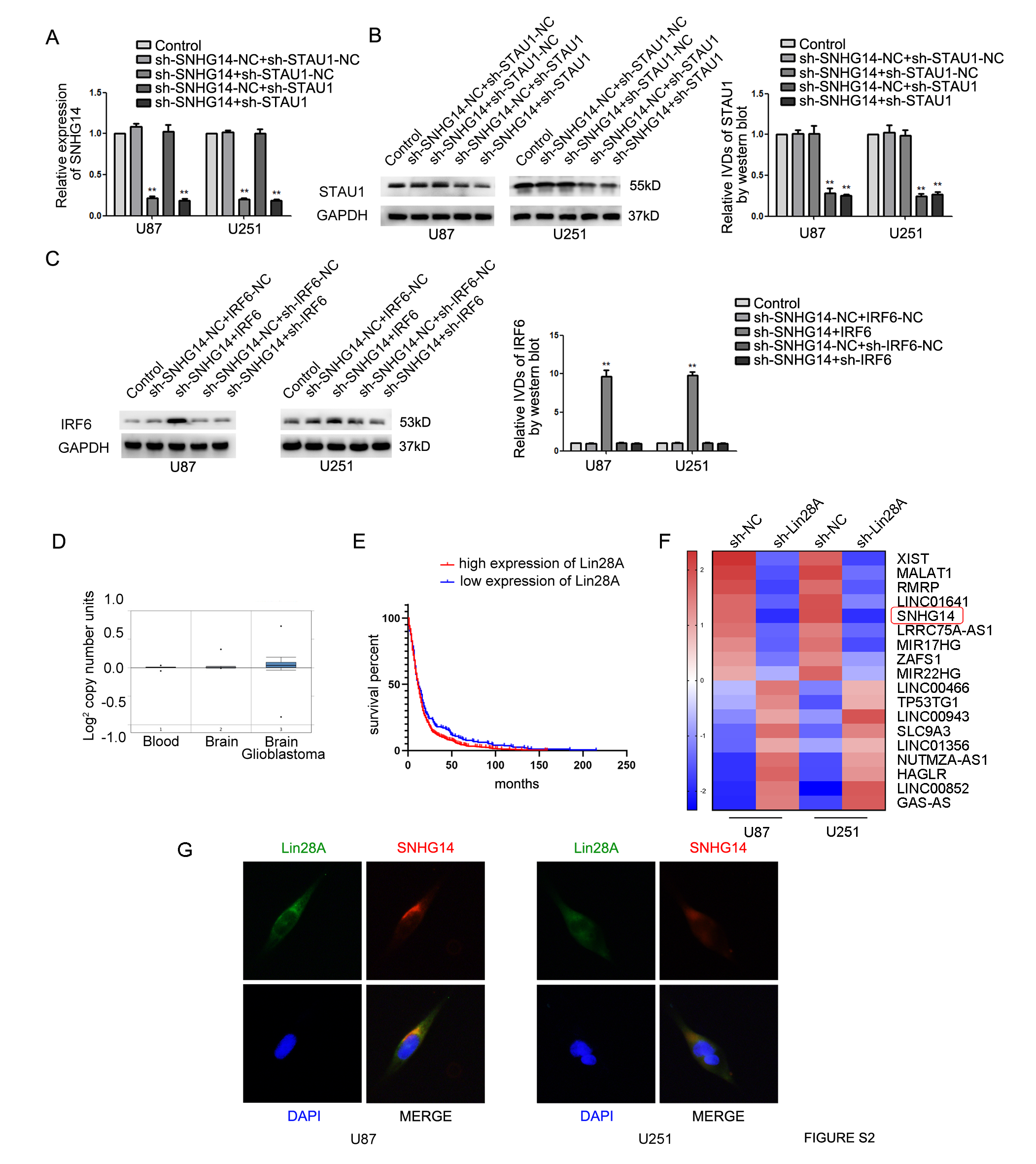

Supplement: Supplementary file 3 — Supplymentary figure 2 [file 41419_2020_2650_MOESM3_ESM.tif]

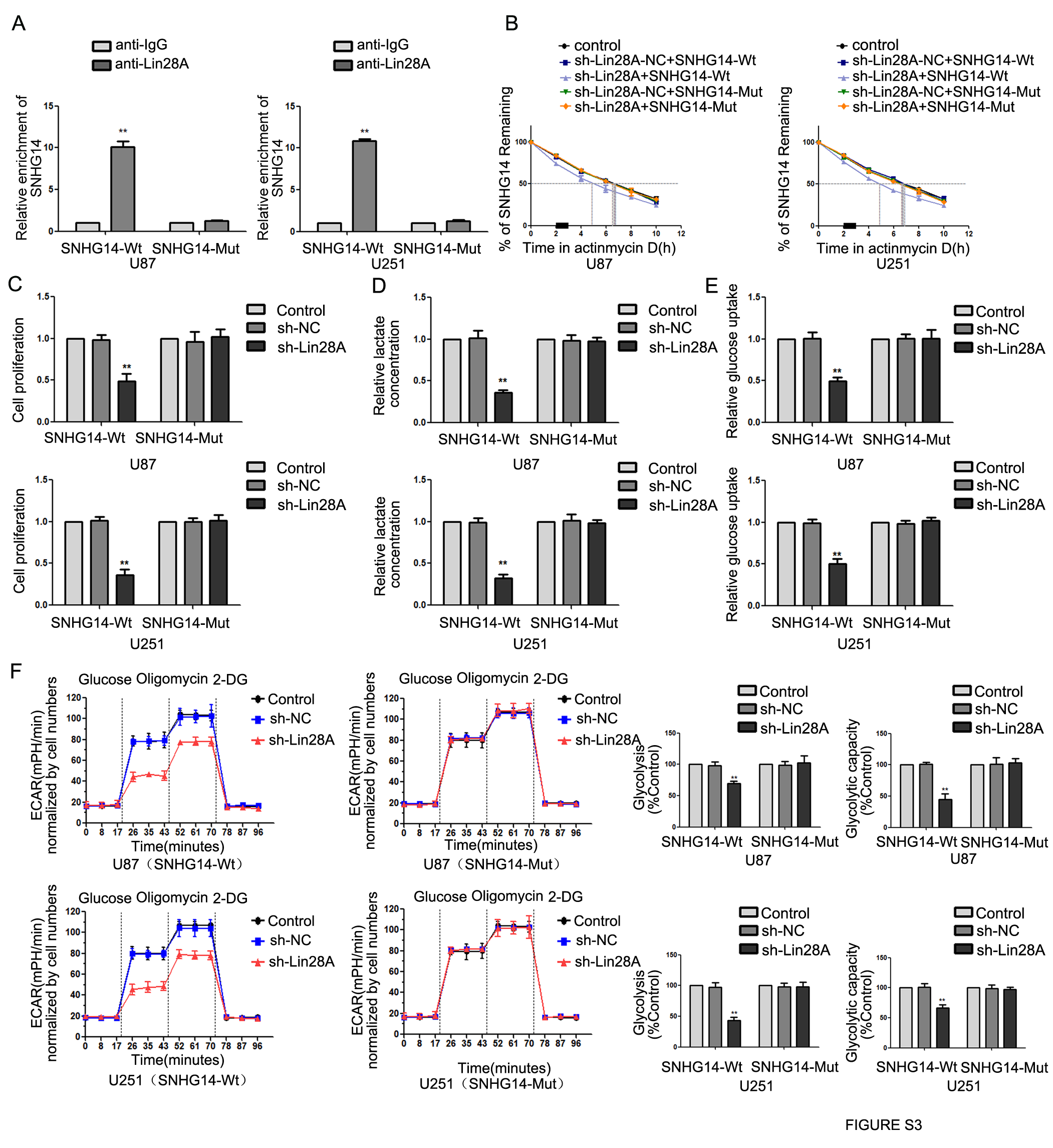

Supplement: Supplementary file 4 — Supplymentary figure 3 [file 41419_2020_2650_MOESM4_ESM.tif]

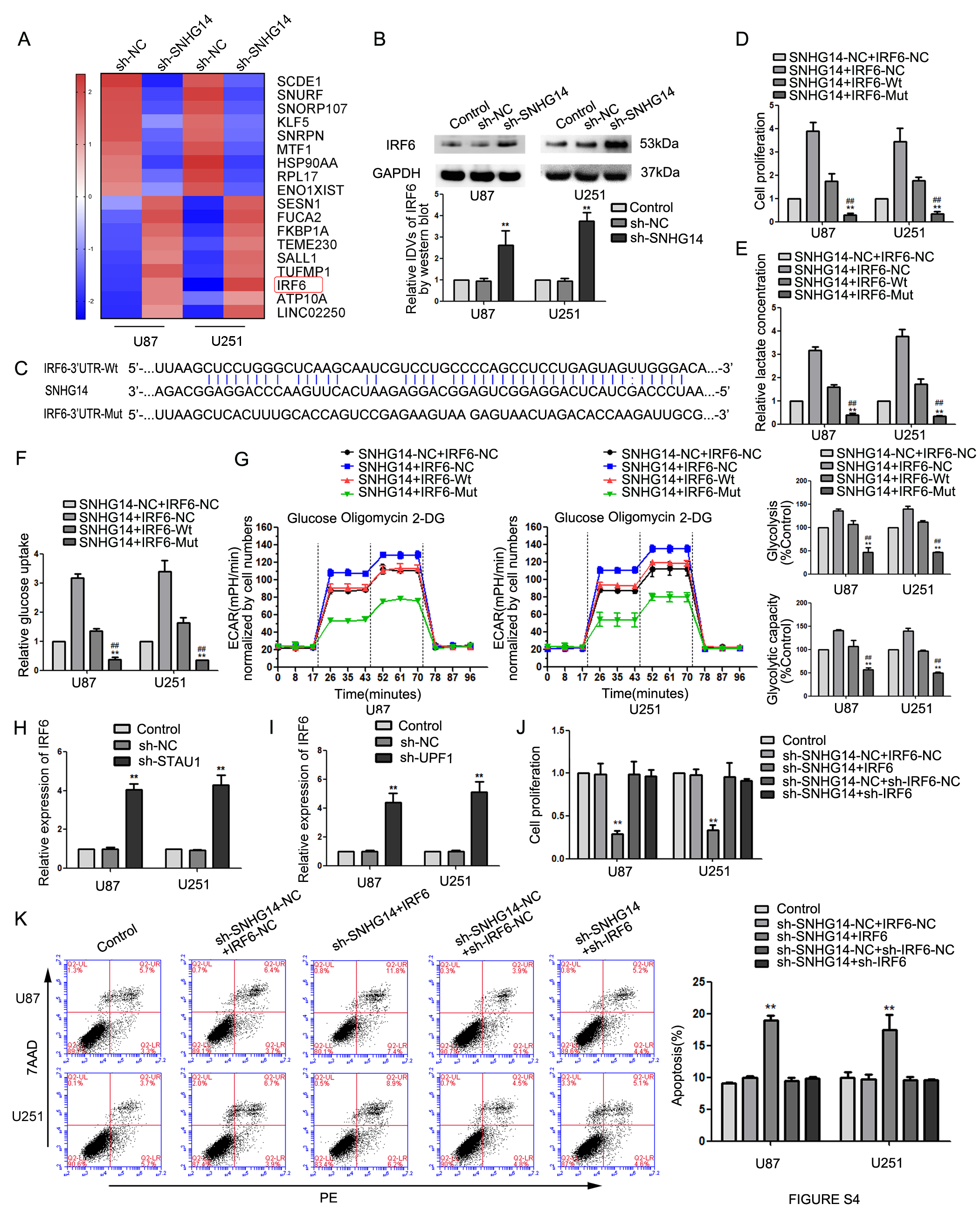

Supplement: Supplementary file 5 — Supplymentary figure 4 [file 41419_2020_2650_MOESM5_ESM.tif]

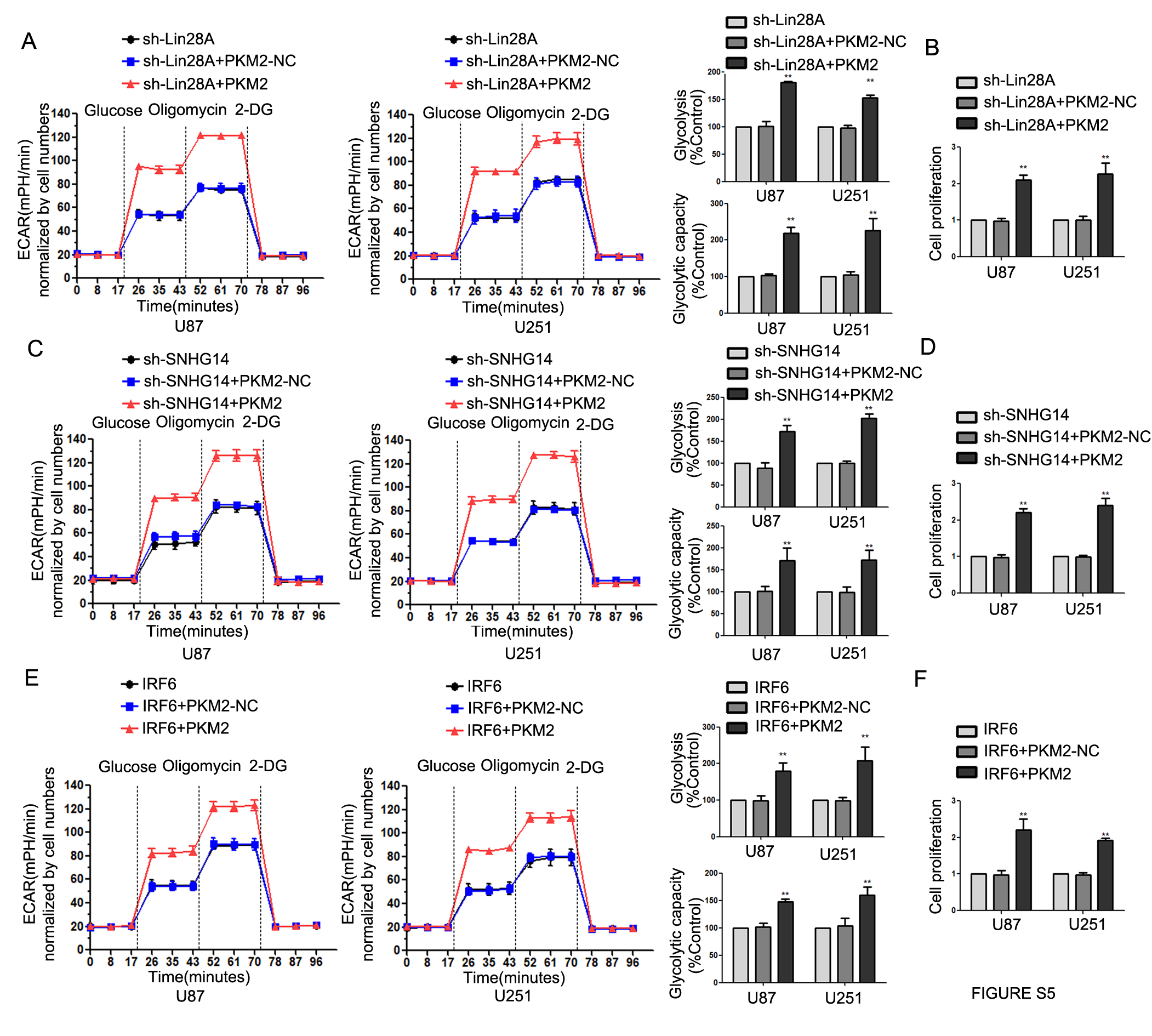

Supplement: Supplementary file 6 — Supplymentary figure 5 [file 41419_2020_2650_MOESM6_ESM.tif]
