# Supplementary material for: Lin28A promotes IRF6-regulated aerobic glycolysis in glioma cells by stabilizing SNHG14
Source: Cell Death Dis. 2020 Jun 11;11(6):447. doi: 10.1038/s41419-020-2650-6 (PMC7289837; doi:10.1038/s41419-020-2650-6)
Supplement: Supplementary file 7 — Supplymentary figure legends [file 41419_2020_2650_MOESM7_ESM.doc]

**Supplyment Fig. 1.** Transfection efficiency. (A,B) Expression of Lin28A by qRT-PCR and western blot when depleting Lin28A. (C,D) Expression of Lin28A by qRT-PCR and western blot after upon re-expressing Lin28A in sh-Lin28A cells. (E) Expression of SNHG14 by qRT-PCR after depleting SNHG14. (F) Expression of SNHG14 by qRT-PCR upon re-expressing SNHG14 in sh-SNHG14 cells. (G) Expression of SNHG14 when depleting Lin28A and SNHG14. (H,I) Expression of Lin28A by qRT-PCR and western blot when depleting Lin28A and SNHG14. (J,K) Expression of IRF6 by qRT-PCR and western blot upon over-expressing or silencing IRF6. (L,M) Expression of STAU1 by qRT-PCR and western blot when depleting STAU1. (N,O) Expression of UPF1 by qRT-PCR and western blot when depleting UPF1. Data are presented as mean±SD (n=3, each group). **P*<0.05, ***P*<0.01 versus empty vector group. Using one-way analysis of variance for statistical analysis.

**Supplyment Fig. 2.** (A) Expression of SNHG14 by qRT-PCR when depleting SNHG14 and STAU1. (B) Expression of STAU1 by western blot when depleting SNHG14 and STAU1. (C) Expression of IRF6 by western blot under condition of sh-SNHG14+IRF6 or sh-SNHG14+sh-IRF6. (D) Expression of Lin28A in glioblastoma samples was analyzed by oncomine database. Lin28A was significantly high expressed in glioblastoma (n=582). **P<*0.05 versus brain group (n=37). (E) Overall survival curve according to oncomine database. Higher expression of Lin28A contributed to shorter survival time (*P*=0.0195). (F) RNA microarray was performed to detect the differential gene when Lin28A was knockdown. (G) Fluorescence *in situ* hybridization analysis of the subcellular location of SNHG14 and Lin28A in U87 and U251 cells.

**Supplyment Fig. 3.** (A) The enrichment of SNHG14 in Lin28A immunoprecipitation group in SNHG14-Wt or SNHG14-Mut group. Data are represented as mean±SD (n=3, each group). ***P*<0.01 versus anti-IgG group, using Student’s t test. (B) T_1/2_ of SNHG14 was detected upon depleting of Lin28A in SNHG14 wild-type or SNHG14 mutant cells. Cell proliferation (C), lactate production (D), glucose uptake (E) and ECAR (F) were detected upon depleting Lin28A in SNHG14 wild-type or SNHG14 mutant cells.Data are presented as mean±SD (n=3, each group). ***P*<0.01 versus sh-NC group. Using one-way analysis of variance for statistical analysis.

**Supplyment Fig. 4.**(A) RNA microarray was performed to detect the differential gene by silencing SNHG14. (B) Expression of IRF6 by western blot upon depleting SNHG14. (C) Predicted by bioinformatics database (IntaRNA), the sequence might bind SNHG14 to IRF6 mRNA 3’UTR(IRF6-Wt) and a generated mutant sequence (IRF6-Mut) was generated. (D) Cell Counting Kit-8 (CCK-8) assay was applied to evaluate the proliferation induced upon wild-type or mutation of 3’-UTR of IRF6. Lactate production (E), glucose uptake (F) and ECAR (G) were aslo measured. Data are presented as the mean±SD (n=3 in each group). ***P*<0.01 versus SNHG14-NC+IRF6-NC group. (H) Expression of IRF6 by qRT-PCR upon depleting STAU1. Data are presented as the mean±SD (n=3 in each group). ***P*<0.01 versus sh-NC group. (I) Expression of IRF6 by qRT-PCR upon depleting UPF1. Data are presented as the mean±SD (n=3 in each group). ***P*<0.01 versus sh-NC group. (J) CCK-8 assay was applied to evaluate the proliferation upon the regulation of SNHG14 and IRF6. (K) Flow cytometry analysis applied to evaluate apoptosis ability upon the regulation of SNHG14 and IRF6. Data are presented as the mean±SD (n=3 in each group). ***P*<0.01 versus sh-SNHG14-NC+IRF6-NC group. Using one-way analysis of variance for statistical analysis.

**Supplyment Fig. 5.** ECAR and cell proliferation were detected to eveluate the role of over-expressing PKM2 in sh-Lin28A or sh-SNHG14 or IRF6 cell lines. Data are presented as the mean ± SD (n=3, each group), ***P*<0.01 versus empty vector group. Using one-way analysis of variance for statistical analysis.
